# Correction: Randomized nutrient bar supplementation improves exercise-associated changes in plasma metabolome in adolescents and adult family members at cardiometabolic risk

**DOI:** 10.1371/journal.pone.0294377

**Published:** 2023-11-09

**Authors:** Michele Mietus-Snyder, Nisha Narayanan, Ronald M. Krauss, Kirsten Laine-Graves, Joyce C. McCann, Mark K. Shigenaga, Tara H. McHugh, Bruce N. Ames, Jung H. Suh

In the original Tables 5 and 6 of this article [1], the *p*-values had been erroneously transcribed from the Pairwise Comparisons section of the SPSS data output. In addition, the threshold for significant within group changes before and after intervention is incorrectly denoted as *p*≤0.002 in the figure legend; the correct threshold is *p*<0.05. The corresponding corrected *p*-values and figure legends have been updated in Tables 5 and 6, as shown below. To clarify the meaning of Pairwise Diff and Time x group, the following abbreviation is also added to the legend of both tables: Pairwise Diff-within group pre-post mean change.

In Table 5, when the correct *p*-values are used, there is only a significant time by group difference between Control and Intervention groups for sphinganine, the one sphingoid base that was not originally included in this Table. The results for sphinganine are already discussed in the text and have been added to the corrected table.

**Table 5:** Intervention effects on plasma sphingolipid bases.

	CONT			INT			
Sphingoid Bases	Baseline mean ± SE (nmol/L)	Pairwise Diff (nmol/L)	95% CI (nmol/L)	Baseline mean ± SE (nmol/L)	Pairwise Diff (nmol/L)	95% CI (nmol/L)	Time x group *p* values
Sphinganine	76.9 ± 8.4	**+43.7** *	+16.6 - +70.9	81.9 ± 12.7	-13.1	-44.7 - +18.5	0.007
Sphingosine	38.2 ± 4.7	**+19.4** *	+1.0 - +37.8	241.9 ± 82.7	-10.2	-95.1 - +74.7	0.5
Dihydro-sphingosine-1-phosphate	5.2 ± 0.7	+2.4	0.0 - + 4.7	4.2 ± 0.4	+1.3	-0.3 - + 3.0	0.5
Sphingosine -1-phosphate	282.9 ± 25.1	**+88.8** *	+0.4 - +177.1	256.8 ± 22.7	**+130.5** *	+38.4 - +222.5	0.5

*denotes significant within group changes (bolded) before and after intervention at *p*<0.05

SE = standard error, Time x group denotes significance of between group changes

CI-confidence interval of within group changes, Pairwise Diff-within group pre-post mean change

In Table 6, the corrected *p*-values indicate that there is no longer a significant change between groups in aromatic amino acids, but the other differences in gluconeogenic, sulfur redox and urea cycle intermediates as well as in the Arginine Bioavailability Index still stand. In addition, minor differences in the baseline values reported in Table 6 are due to the inclusion of baseline metabolomic amino acid data on two participants who had dropped out early from the study together with their full family units, and so were not included in any other reported analyses, nor in the study data set. The baseline data for Table 6 have accordingly been updated together with the correction of table *p*-values.

**Table 6:** Differential changes in plasma amino acid metabolites following CONT and INT interventions.

	Control			INT			
Amino Acids	Baseline mean ± SE (µmol/L)	Pairwise Diff (µmol/L)	95% CI (µmol/L)	Baseline mean ± SE (µmol/L)	Pairwise Diff (µmol/L)	95% CI (µmol/L)	Time x Group
Arginine	83.5 ± 3.7	-7.1	-16.6 - + 2.40	81.7 ± 4.1	+10.3 SD	-3.5–24.1	0.042
Serine	128.4 ± 9.5	+7.0	-14.8 - +28.9	159.8 ± 9.7	**-36.2** *	-53.3 - -19.2	0.002
Proline	188.4 ± 9.7	+22.0	-0.07 - +44.1	253.8 ± 12.6	**-54.7** *	-76.5 - -32.9	<0.001
Aspartate	25.1 ± 2.2	+2.2	-3.3 - +7.6	39.4 ± 2.5	**-11.6** *	-17.2 - -6.0	0.001
Cystathionine	1.0 ± 0.2	-0.07	-0.2 - +0.1	0.81 ± 0.04	**-0.27** *	-0.36 - -0.19	0.037
Sarcosine	19.4 ± 2.3	-1.8	-7.4 - +3.8	30.2 ± 1.7	**-10.7**	-14.6 - -6.8	0.011
Ornithine	82.1 ± 8.3	**+44.5** *	+13.0 - +75.9	124.6 ± 7.9	+5.9	-38.4 - +50.2	0.165
Arg Bioavail Ratio	0.65 ± 0.06	**-0.20** *	-0.31 - -0.07	0.41 ± 0.03	**+0.26** *	+0.05 - +0.5	<0.001
Lysine	246.2 ± 12.5	+37.7	-0.66 - +76.0	308.1 ± 12.1	-7.1	-30.9 - +16.6	0.051
Alanine	226.7 ± 13.4	**+35.1** *	+7.0 - +63.2	293.7 ± 16.5	**-50.3** *	-77.8 - -22.8	<0.001
Glutamine	544.2 ± 30.5	-24.1	93.7 - +45.5	604.1 ± 26.1	**-99.4** *	-149.2 - -49.7	0.084
Threonine	175.7 ± 15.7	**+31.3** *	+0.14 - +62.6	181.0 ± 10.2	-12.6	-37.5 - +12.3	0.031
Methionine	25.3 ± 1.4	-0.60	3.2 - +2.0	28.1 ± 1.1	**-3.3** *	-5.1 - -1.5	0.094
Fischer Ratio	1.9 ± 0.08	**+0.13** *	+0.005 - +0.26	1.7 ± 0.06	**+0.2** *	+0.05 - +0.35	0.52
Citrulline	60.1 ± 5.5	+11.8	-6.2 - +29.7	89.4 ± 10.2	**-33.2** *	-59.1 - -7.2	0.005
Histidine	20.9 ± 1.1	+1.4	-0.45 - +3.3	19.3 ± 1.1	**+2.6** *	+0.39 - +4.9	0.412
Tryptophan	39.7 ± 3.2	+5.3	-1.2 - +11.7	49.1 ± 2.2	+1.6	-3.0 - +6.2	0.37
Leucine	64.8 ± 4.0	**+10.2** *	+4.1 - +16.2	73.1 ± 2.5	+0.16	-6.4 - +6.8	0.029
Phenylalanine	28.2 ± 1.8	+4.2	-1.3 - +9.6	36.6 ± 1.1	-4.5	-12.1 - +3.1	0.07

*denotes significant within group changes before and after intervention at *p*<0.05

Time x Group denotes significance of between group changes

CI-confidence interval of within group changes, Fischer Ratio-ratio of sum of branched chain amino acids (valine, leucine, isoleucine) over sum of aromatic amino acids (phenylalanine, tyrosine), Arg Bioavail Ratio-ratio of arginine to sum of citrulline and ornithine, Pairwise Diff-within group pre-post mean change

Statements describing Tables 5 and 6 in the manuscript are updated as follows:

- The fourth sentence in the Results section of the Abstract is corrected to: Nutrient bar supplementation (INT) blunted the rise in sphinganine and significantly decreased ureagenic and gluconeogenic amino acid metabolites.
- The second sentence in the third paragraph of the Metabolomic and lipidomic changes observed with intervention subsection of the Results is corrected to: In CONT only, levels of sphinganine increased significantly by 59%, resulting in significant pairwise differences between CONT and INT groups (Table 5).
- The third sentence in the fourth paragraph of the Metabolomic and lipidomic changes observed with intervention subsection of the Results is removed.
- The fifth paragraph of the Metabolomic and lipidomic changes observed with intervention subsection of the Results is corrected to: Table 6 lists amino acid metabolites with significant within group and between group changes. Results show that among CONT, fasting levels of ornithine, alanine, threonine and leucine increased. In contrast, the nutrient bar INT altered amino acid metabolism, such that concentrations of these gluconeogenic, sulfur redox and urea cycle intermediates were blunted or significantly decreased. There were also significant reductions in the INT group in serine, proline, aspartate, cystathionine, glutamine, and methionine. Time by group changes were significantly different for arginine, serine, proline, aspartate, cystathionine, sarcosine, alanine and citrulline. The arginine bioavailability ratio also went down in CONT and up significantly in the INT group with a significant time by group comparison.
- The third sentence of the sixth paragraph of the Discussion is corrected to: In support of this mechanism, we also observed significant lowering of non-essential amino acids (NEAA), serine, proline, aspartate, and alanine (Table 6).
- The first sentence of the eighth paragraph of the Discussion is corrected to: In both CONT and INT participants, sphingosine-1-phosphate (S1P) levels increased.

There are also errors in Table 2. Physical activity score for Teen Controls post intervention is incorrectly denoted as significantly different; the asterisk in the fourth row of the fifth column is removed. Also, significant Time x Group interactions for WHRatio and HR for PAC are not correctly denoted. “**P” is added to the eighth and twenty-second rows of the first column. The third line of the legend is corrected to: ** Denotes statistically significant (p < 0.05) time by group interactions for the designated variable for both PAC and Teens (Vitamin D levels) unless specified (P for PAC only, WHRatio and HR; T for Teens only, SBP). Please see the correct Table 2 below.

**Table 2:** Effects of Interventions on Anthropometric and Clinical Measures.

	CONTROL				INTERVENTION			
	PAC		Teen		PAC		Teen	
	Baseline	Post	Baseline	Post	Baseline	Post	Baseline	Post
Activity Score	8.6 (6.9)	14.8 * (2.8)	12.1 (4.9)	14.3 (4.0)	8.2 (3.6)	10.6 * (3.5)	10.3 (4.2)	14.3* (3.7)
Vit. D (nmol/L)**	37.4 (10.0)	40.6 (8.5)	49.7 (21.2)	57.2 (18.4)	47.9 (14.2)	74.4 (26.7)*	53.4 (16.2)	72.6 (24.7)*
Weight (Kg)	103.7 (14.9)	103.6 (15.4)	102.9 (27.9)	103.9 (28.6)	82.9 (18.6)	83.2 (18.7)	85.3 (8.7)	86.1 (8.8)
Waist Circ (cm)	115.9 (9.1)	110.5 * (10.7)	106.9 (19.3)	106.6 (18.3)	102.0 (16.9)	101.5 (13.7)	103.0 (10.3)	103.1 (9.60
WHRatio**P	0.71 (0.06)	0.67* (0.07)	0.63 (0.10)	0.63 (0.08)	0.63 (0.11)	0.63 (0.10)	0.64 (0.09)	0.63 (0.08)
BMI (kg/m^2^)	38.6 (5.2)	38.5 (5.1)	35.9 (8.1)	36.1 (8.5)	31.8 (5.9)	31.9 (6.0)	32.2 (4.6)	32.5 (4.8)
Adipo (ng/ml)	1556 (1389)	1933 (1738)	1313 (588)	1212 (433)	2506 (1654)	3134 (2584)	1296 (587)	1193 (451)
CRP (nmol/L)	43.8 (25.7)	59.1 (51.4)	57.1 (30.5)	34.3 (25.7)	31.4 (16.2)	41.0 (29.5)	27.6 (22.8)	34.3 (23.8)
Glucose (nmol/L)	5.5 (0.2)	5.6 (0.5)	5.3 (0.3)	5.4 (0.5)	5.4 (0.4)	5.4 (0.4)	5.1 (0.3)	5.2 (0.5)
Insulin (pmol/L)	21.1 (13.5)	18.4 (8.8)	29.9 (21.2)	29.4 (15.9)	12.8 (4.6)	12.5 (7.4)	25.8 (12.8)	29.5 (21.9)
HOMA	5.3 (3.6)	4.6 (2.1)	7.2 (5.5)	7.2 (4.1)	3.1 (1.3)	3.1 (2.0)	5.9 (3.0)	6.8 (5.2)
TC (mmol/L)	5.1 (1.1)	5.0 (1.)	4.2 (0.8)	4.1 (0.9)	5.2 (0.5)	5.4 (0.6)	4.5 (1.0)	4.6 (1.0)
TG (mmol/L)	1.3 (0.6)	1.3 (0.6)	1.4 (0.9)	1.5 (1.1)	2.0 (1.8)	1.7 (0.9)	1.2 (0.4)	1.3 (0.6)
LDL (mmol/L)	3.2 (1.0)	3.1 (1.3)	2.5 (0.6)	2.3 (0.6)	3.0 (0.4)	3.1 (0.4)	2.8 (0.9)	2.7 (0.7)
HDL (mmol/L)	1.3 (0.3)	1.3 (0.3)	1.1 (0.2)	1.1 (0.2)	1.4 (0.3)	1.4 (0.2)	1.2 (0.3)	1.2 (0.)3
TGHDLR	2.4 (1.9)	2.5 (1.7)	2.9 (2.1)	3.5 (2.9)	4.3 (6.3)	2.9 (2.2)	2.5 (1.1)	2.6 (1.1)
SBP **T (mm Hg)	127 (15)	124 (15)	112 (8)	117 (9)	120 (11)	119 (14)	117 (8)	109* (10)
DBP (mm Hg)	77 (9)	80 (9)	66 (7)	65 (7)	79 (10)	76 (8)	65 (9)	65 (8)
HR**P	67 (13)	69 (10)	75 (7)	80 (8)	72 (9)	64 (8)	70 (10)	73 (11)

Values are mean ± SD (standard deviation)

* Denotes statistically significant differences (*p* < 0.05) by paired student’s t-test within a group.

** Denotes statistically significant (*p* < 0.05) time by group interactions for the designated variable for both PAC and Teens (Vitamin D levels) unless specified (P for PAC only, WHRatio and HR; T for Teens only, SBP)

Abbreviations as listed with Table 1.

Statements describing Table 2 in the manuscript are updated as follows:

- The fourth sentence in the first paragraph of the Clinical changes in CVD risk factors following intervention subsection of the Results is corrected to: Self-report activity increased in all but teen controls (Table 2).
- The second sentence in the fourth paragraph of the Discussion is corrected to: In this trial, despite excellent participation in weekly exercise sessions, self-report of significantly increased exercise between sessions in all but teen controls, and both subjective and objective evidence of compliance with nutrient bar intake in the INT group, there were minimal changes in traditional parameters of dyslipidemia, inflammation, and insulin resistance (Table 2).

There is an error in the second sentence in the first paragraph in the Baseline correlations between metabolomic profiles and clinical biomarkers section of the Results, since obesity was an inclusion criterion for Teens, not PAC (parent adult carer). The correct sentence is: Most participants in this study, both Teens and PACs met criteria for obesity and had baseline CRP > 14.3 nmol/L. The same error exists in the first sentence in the Conclusion section of the Abstract. The correct sentence is: Nutrient bar supplementation with increased physical activity in Teens with obesity and PAC elicits favorable metabolomic changes that correlate with improved dyslipidemia.

These corrections do not alter the main findings reported nor do they change the conclusion: nutrient bar supplementation with increased physical activity in adolescents and adult caretakers with obesity elicits favorable metabolomic and lipidomic changes that correlate with improved dyslipidemia.

As per standards for using patient-first language, the use of the words overweight and obese as adjectives is corrected as follows:

- The first sentence of the Methods section of the Abstract is corrected to: Predominantly minority and female adolescents (Teens) with obesity/parent adult caretaker (PAC) units were recruited from a pediatric obesity clinic.
- The first sentence in the Conclusion section of the Abstract is corrected as above.

Concerns were raised about the statistical approach used for group comparisons and whether a mixed model approach would be more appropriate. The authors clarify that the intention of this analysis was to identify any differences in baseline cardiometabolic profiles. The family units in the inner-city cohort were not all traditional units.

The following is hereby added to the article’s Acknowledgements: We are grateful to Drs. Jasmine Jamshidi-Naeini and Colby Vorland, investigators from the research group of Dr. David Allison, Indiana University-Bloomington School of Public Health, who brought the corrections in Tables 2, 5 and 6 to our attention in the course of performing their own analysis of the study data set accounting for the clustering and nesting effects of household units, an analysis that validated our published study conclusions.

A member of *PLOS ONE’*s Statistical Advisory Board reviewed this correction to ensure that the Results and Conclusions of the published article are supported.
