# Supplementary material for: Randomized nutrient bar supplementation improves exercise-associated changes in plasma metabolome in adolescents and adult family members at cardiometabolic risk
Source: PLoS One. 2020 Oct 20;15(10):e0240437. doi: 10.1371/journal.pone.0240437 (PMC7575082; doi:10.1371/journal.pone.0240437)
Supplement: S1 Protocol — (PDF) [file pone.0240437.s001.pdf]

CHILDREN'S HOSPITAL
& RESEARCH CENTER OAKLAND

Institutional Review Board
Application for Study Review

Study Information	
**IRB Number:**	
**Protocol Title:**	The Impact of a Nutritional Supplement on Weight and Metabolic Health in a Parent-Child Intervention
**Principal Investigator:**	Bruce Ames, PhD
Department:	CHORI Nutrition and Metabolism Center
Phone:	510 450-7625-
Fax:	510 597-7128-
E-mail:	bames@chori.org
**Co-Investigator:**	Michele Mietus-Snyder
Department:	CHORI Nutrition and Metabolism Center
Address (if outside CHRCO):	CNMC address: Obesity Institute, 2.5 WW, 111 Michigan Avenue, NW Washington, DC 20010
Phone:	(415) 640-5285<br>(202) 476-5161
Fax:	(202) 476-3470
E-mail:	mmsnyder@cnmc.org
**Co-Investigator:**	Ash Lal- Primary Clinical Contact on site for study
Department:	CHORI Nutrition and Metabolism Center
Phone:	(510) 428-3264 (Pager 510-539-5261)
Fax:	**(510) 597-7128**
E-mail:	**alal@mail.cho.org**
**Co-Investigator:**	Kirsten Laine-Graves
Phone:	(415) 606-1461
Fax:	(510) 597-7128
E-mail:	kgraves@chori.org Also: kirstenlaineg@yahoo.com
**Study Coordinator:**	To be named
Phone:	(415) 428-3885 x5061
Fax:	(510) 597-7128
**Primary Contact Person:**	Michele Mietus-Snyder

CHILDREN'S HOSPITAL
& RESEARCH CENTER OAKLAND

Funding	
☐ Federal ☐ Pharmaceutical Company ☒ Private Foundation ☐ Internal Grant Program<br>☐ Industry ☐ Children's Oncology Group (COG) ☐ Other, Specify:	
**Funding Source:**	Bruce and Giovanna Ames Foundation
**Contract or Grant Title:**	
**Contract or Grant #:**	
**Address of Sponsor:**	CHORI
**Contact Person:**	Brad Barber
**Phone:**	(510) 428-3813
**E-mail:**	

Subject Category	
☒	Subjects admitted strictly for research purposes. Hospitalization and laboratory costs are paid by the funding source.
☐	Research subjects receiving established medical care. Hospitalization and laboratory costs paid by third party. (except for tests performed exclusively for the study)
☐	Research subjects admitted on an industry-sponsored protocol. All costs paid by industry sponsor. Requires $2,200 IRB application and set-up fee.

ClinicalTrials.Gov Registration	
Does this study need to be registered on www.clinicaltrials.gov? (see below)	☒ No ☐ Yes

General Requirements
U.S. Public Law 110-85 (Food and Drug Administration Amendments Act of 2007 or FDAAA), Title VIII, Section 801 mandates that a "responsible party" (i.e., the sponsor or designated principal investigator) register and report results of certain "applicable clinical trials":

- **Trials of Drugs and Biologics:** Controlled, clinical investigations, other than Phase I investigations, of a product subject to FDA regulation;
- **Trials of Devices:** Controlled trials with health outcomes of a product subject to FDA regulation (other than small feasibility studies) and pediatric post-market surveillance studies.

"Applicable clinical trials" generally include interventional studies (with one or more arms) of drugs, biological products, or devices that are subject to FDA regulation, meaning that the trial has one or more sites in the U.S, involves a drug, biologic, or device that is manufactured in the US (or its territories), or is conducted under an investigational new drug application (IND).

CHILDREN'S HOSPITAL
& RESEARCH CENTER OAKLAND

Investigator's Assurance
The Principal Investigator must assure the IRB that all procedures performed under the project will be conducted in strict accordance with all applicable federal, state and local regulations and laws regarding the protection of human subjects in research including, but not limited to:

- Use of qualified personnel to conduct the project according to the protocol approved by the IRB.
- Ensuring that no changes are made to the approved protocol or consent form without prior IRB approval (except in an emergency to safeguard the well-being of subjects).
- Using the most current, approved, IRB stamped consent form to obtain informed consent from subjects or their legally responsible representative.
- Prompt reporting to the IRB in writing of any changes in research activity, unanticipated problems involving risks to subjects or others, and adverse events (AEs), within the time period specified by IRB policy.
- If I will be unavailable to direct this research personally, as when on leave or vacation, I will arrange for a co-investigator to assume direct responsibility in my absence. If this is not a co-investigator named in my absence, I will notify the IRB in writing of the responsible party.

Michele Mietus-Snyder — April 8, 2011

Principal Investigator's Signature — Date

Faculty Sponsor's Assurance (if applicable)
By my signature as sponsor on the research application, I certify that the student/investigator listed on page one is knowledgeable about the regulations and policies governing research with human subjects and has sufficient training and experience to conduct this particular study in accord with the approved protocol. In addition,

- I agree to meet with the student/investigator on a regular basis to monitor study progress.
- Should problems arise in the course of the study, I agree to be available to personally supervise the student in solving them.
- I assure that the student/investigator will promptly report Adverse Events to the IRB according to the schedule indicated above.
- If I will be unavailable, as on vacation, I will arrange an alternate faculty sponsor to assume responsibility during my absence and I will advise the IRB of such arrangements.

______________________________ ______________

Signature of Faculty Sponsor — Date
(if co-investigator is a student, resident, or fellow)

CHILDREN'S HOSPITAL
& RESEARCH CENTER OAKLAND

Statement of Financial Interests

By the signatures below, each investigator is certifying that either no financial interest exists or a complete listing of all financial interests related to the proposed project is provided. All individuals named below further acknowledge their responsibility to disclose any new reportable financial interest obtained during the term of the project. The Principal Investigator's signature also certifies that all individuals required to make disclosures have been listed below: (Attach additional sheet if necessary)

Do you, your spouse, or dependent children, have a financial interest in the work to be conducted under the proposed project?

[signature] 4/8/11 ☒ No ☐ Yes: Attach Financial Disclosure
Signature of Principal Investigator Date

____________________________ ________ ☐ No ☐ Yes: Attach Financial Disclosure Form
Signature of Co- Investigator Date

____________________________ ________ ☐ No ☐ Yes: Attach Financial Disclosure Form
Signature of Co- Investigator Date

____________________________ ________ ☐ No ☐ Yes: Attach Financial Disclosure Form
Signature of Co- Investigator Date

____________________________ ________ ☐ No ☐ Yes: Attach Financial Disclosure Form
Signature of Co- Investigator Date

Documentation of Investigator Education in Human Subject Research

Training in Human Research Subject Protections is required for all individuals who are participating in research activities at Children's Hospital & Research Center Oakland and Children's Hospital Oakland Research Institute. The Principal Investigator, Co-Investigators and other study staff interacting with research subjects must complete the University of Miami School of Medicine CITI Program in the Protection of Human Research Subjects to obtain IRB approval of a new study. Core Modules (Basic Course) must be completed only once, and Continuing Education Modules (Refresher Course) must be completed annually. (Attach additional sheet if necessary for other study personnel.)

B. Ames ☒ Basic/Refresher CITI Course Completed

Name of Principal Investigator

Michele Mietus-Snyder ☒ Basic/Refresher CITI Course Completed

Name of Co-Investigator

Ash Lal ☐ Basic/Refresher CITI Course Completed

Name of Co-Investigator

Kirsten Laine-Graves ☒ Basic/Refresher CITI Course Completed

Name of Co-Investigator

CHILDREN'S HOSPITAL
& RESEARCH CENTER OAKLAND

Summary Information
**1. Age Range of Eligible Subjects:** 14-70 yr
**2. Subject Population:** (Please check all that apply)

☐	a. neonates	☒	f. minors	☐	k. cancer patients
☒	b. minorities/immigrants	☒	g. normal volunteers	☐	l. terminally ill
☐	c. comatose patients	☐	h. institutionalized	☐	m. students
☐	d. decisionally impaired	☐	i. wards of the court	☐	n. pregnant women
☐	e. elderly	☐	j. prisoners or parolees	☒	o. Overweight/Obese children

3. Study Type: If the research involves any of the following, please check all that apply.

☐	a. Investigational Drug (IND)	☐	l. Investigational Device (IDE – HUD)
☒	b. Genetic Research (DNA)	☐	m. Vaccine Trial
☒	c. Collection of Biological Specimens for Banking and/or Collection of PHI (identified data) for a Database	☐	n. Transplantation
☐	d. Collection of Remnant Surgical Specimens	☒	o. PI or Co-PI is the treating clinician
☐	e. Magnetic Resonance Imaging (MRI)	☐	p. Radiation (including X-ray, DXA)
☐	f. Gene Transfer Therapy	☐	q. Biohazardous Waste
☐	g. HIV Screening	☐	r. HIV/AIDS Research
☐	h. Alcohol and Drug Abuse Research	☐	s. Controlled Substances
☐	i. Acute Care Waiver of Informed Consent	☐	t. Deception
☐	j. Behavioral Observations	☐	u. Audio/Videotapes or Focus Groups
☒	k. Surveys, Questionnaires or Psychological Testing		

4. Signature Page: The signature page and study packet should go directly to those whose signature is required, not to the IRB office.

CHILDREN'S HOSPITAL
& RESEARCH CENTER OAKLAND

SIGNATURE PAGE

Print this page and circulate with the study submission packet, including the expenses worksheet (budget)

IRB #: _____ **Principal Investigator: _____**

Study Title:

Return Initialed Form to (study coordinator): _____ Ext. _____

The following individuals must review your completed application, and initial this page, before it can be approved by the CHRCO IRB:

CHORI STAFF (*Please circulate* ***a single copy*** *to CHORI Staff*)

1. Senior Vice President, Research, Alexander Lucas, PhD ___________
2. Vice President, Research Administration, Kathleen Hogue Gonzalez, CRA ___________
3. Research Counsel, Suzanne Haendel, JD (*if there is a contract with a sponsor*) ___________ N/A ☐

**CHORI Front Office Use Only:** Received on: _______________	
Logged In: ____Yes ____ No	By: _______________
Returned Date: _______________	By: _______________

* *

HOSPITAL STAFF

4. Director of CHRCO Pharmacy, Patrick Fleming, PharmD (if pharmacy resources will be used) ___________ N/A ☒
5. Other CHRCO resources, (as applicable), e.g., clinical laboratory, diagnostic imaging (print name & dept. below) N/A ☐

   ____

   ____
6. Administrative Director CRC, Laurie Schumacher, Ph.D., MPH (if utilizing CTSI-PCRC resources) ___________ N/A ☐

5. **Data Safety Monitoring:** ***All interventional studies involving <u>greater than minimal risk</u> must include a Data Safety Monitoring Plan (DSMP).*** A DSMP is a plan established to assure that each research study has a system for appropriate oversight and monitoring of the conduct of the study to ensure the safety of participants and the validity and integrity of the data. The DSMP should indicate specifically whether there will be a formal Data Safety Monitoring Board (DSMB) or Data Monitoring Committee (DMC).

Has a Data Safety Monitoring Board been established to review data and/or adverse events related to this study?	☒ N/A (minimal risk)<br>☐ Yes ☐ No

Describe the DSMP below:

6. **Research Sites:** Except for multi-center clinical trials (e.g., industry, COG) list all sites in which the research is to be conducted and attach other IRB approval letters. If applicable, attach letters of support from those institutions. ☐ N/A – Multi-center clinical trial

CHRCO

7. **Investigational drugs/devices:** If any investigational drugs or biologic agents are used in this study, please include two copies of the Investigational Drug Information or Investigator's Brochure (IB) with this application.

☒ **N/A** **IB Number: _____** **Version Date of IB: _____**

Complete and attach the following IRB forms (on the website) as applicable:

Study Review - Investigational Drug Information

Study Review - Investigational Device Information

Protocol Summary

Protocol Version Date: _____ **Amendment #: _____**

Protocol Number: _____ ☐ **N/A (no number)**

Please complete the requested information in the categories below. If the item does not apply to your research, please indicate that the question is not applicable. The information should be intelligible to IRB reviewers from a variety of lay and scientific backgrounds.

8. **Hypothesis:** Briefly explain the hypothesis(es) to be tested. If the study is not designed to test a hypothesis, simply state "None."

Previous studies suggest that micronutrient malnutrition contributes to the disproportionate burden of obesity and cardiometabolic complications of inner city children and their families.

We hypothesize that provision of a comprehensive nutritional supplement CHORI BAR (CB) will enhance adherence to lifestyle modification (on dietary, activity, and stress reduction fronts), and improve weight regulation. Our secondary hypothesis is that formal enrollment of a parent/guardian

with the child will improve both child adherence and outcomes.

9. Purpose of the study: What are the specific scientific aims of this study?

The purpose of this study is to evaluate the efficacy of the CHORI BAR (CB) in an adolescent population The CB formulation with maximum efficacy selected from preliminary studies will be tested in a 2 mo pilot of 32 overweight adolescents (age14-18) enrolled together with one parent to receive a group lifestyle counseling format (led by the Healthy Hearts nutritionist). Participants will be randomized to equally represent insulin resistance based on screening HOMA-IR values in the two groups that will receive this counseling with or without the CB nutrition bar bid. This study design permits close assessment of adherence as bars will be handed out at weekly visits. The enrollment of one parent/guardian with the child participant is also designed to optimize adherence.

a) Characterize at baseline, 2 wk, and 2 mo the physical (BMI, waist circumference, blood pressure), metabolic (glucose and insulin homeostasis), inflammatory indices, micronutrient levels, and behavioral (diet and activity by questionnaire) status in two cohorts obese preadolescents each with one parent/guardian. We aim to enroll 18 dyads in the CB intervention group and 14 in the control group.

b) Evaluate at each of the three timepoints the effect of the CHORI bar above and beyond the effect of standard of care lifestyle counseling and supervised group exercise for weight management on all study measures, the primary outcomes being dyslipidemia, hyperhomocysteinemia, visceral adiposity, inflammation, and insulin resistance.

NB- In all three proposed phases, we will also evaluate novel exploratory assays for gut inflammation, redox status, genomic and mitochondrial DNA integrity, and mitochondrial function that may serve as markers for micronutrient malnutrition and chronic disease risk.

10. Background and Significance: Include a brief summary of previous work that provides a basis for the proposed research and that supports the expectations of obtaining useful information without undue risk to human subjects. **Provide a Bibliography (References)**

This information aids IRB reviewers in assessing how valuable the project is likely to be. If graphs or tables are used to convey information, please maintain a consistent style and make sure that fonts are no less than 11-point in size. If no preliminary data are available, it may be helpful to briefly indicate why this proposed study is a reasonable starting point. Note that some IRB members are non-scientists and may not be familiar with scientific or technical terms.

Optimal nutritional status is associated not only with improved weight and metabolism, but better cognitive function(1), emotional regulation(2), and overall enhanced health quality of life. The United States Department of Agriculture (USDA) MyPyramid guidelines are designed to encourage adequate daily intake of different food groups to achieve optimal macro and micro nutritional goals. We and other find however that recommended servings are rarely met for fiber and nutrient-dense, polyphenolic-rich pigmented vegetables, legumes, whole grains, omega3 PUFA, and low fat dairy, while intake of saturated and trans fats, starchy vegetables and refined carbohydrates exceed recommendations.(3) Even intensive lifestyle counseling programs show more success in changing knowledge than behavior.(4) The Women's Health Initiative Randomized Controlled Dietary Modification Trial aggressively attempted to change women's diets, limiting fat to 20% of total calories and promoting the 5 a day goal for fruit and vegetable intake. Women in the intervention arm made modest strides towards dietary fat reduction but only increased their intake of fresh produce to 1.1 servings/d. The diet not surprisingly, had no significant effect on cardiovascular disease (CVD), though subgroup analysis

revealed a trend toward greater CVD reduction in those who achieved the highest intakes of vegetables and fruit and/or fat specific fat reductions in saturated and trans fats.(5) There are numerous barriers to the universal adoption of proven heart healthy nutritional guidelines and the gaps in nutritional status are abundant, particularly among inner city children (supported by our own data below) due to both financial barriers(6, 7) and time constraints that favor fast food and restaurant dining.(8) Although inner city children enrolled in an intensive lifestyle intervention report improvements in their diets, the change is small, the diets remain poor, and despite weight stabilization, there is limited metabolic improvement (see preliminary data below). We hypothesize that an inexpensive, convenient, nutrient-dense, low calorie dietary supplement can efficiently deliver key nutrients missing from a typical American diet and may prove to be a critical adjunct to intensive lifestyle counseling. If this intervention can satisfy hunger and attenuate inflammation, there will be a favorable impact on metabolism, BMI and adipose distribution, with anticipated benefits to overall self-report quality of life.(9)

Obesity has been associated with suboptimal levels of micronutrients.(10-17) (see also our prelim data below) It remains unclear which comes first, the micronutrient malnutrition or the obesity, but both appear to be interrelated and self-sustaining. It has been postulated that micronutrient deficiencies may increase hunger.(18) Excessive weight gain has also been positively associated with intake of nutrient-poor refined-grain foods.(19) Excess weight alters the absorption, distribution, metabolism, and excretion of micronutrients.(20-22) Micronutrient deficiencies are in turn associated with inflammation(23), (24) and there is evidence that inflammatory biomarkers in normal weight young adults precede and predict accelerated weight gain over the subsequent 3 to 9 years.(25) This relationship also appears to be cyclic as subclinical inflammation is prevalent in obesity and may place an increased demand placed on key vitamins and minerals.(26) Many American adolescent males with adequate macro-nutrient intake of protein, carbohydrate, and fat calories are eating nutrient-depleted foods such that more than 50% consume inadequate amounts of vitamin A and vitamin B6, and more than 75% consume inadequate amounts of fiber, magnesium, phosphorus and zinc(27) (see also C-1).

Human dietary intervention trials and/or mechanistic studies in animals have also demonstrated that diets high in micronutrient-depleted refined carbohydrates and saturated and trans fats are inflammatory.(28) Caloric overnutrition with a Western diet in an animal model triggers inflammatory changes in the hypothalamus that dysregulate normal hunger and satiety mechanisms, contributing to energy imbalance and obesity.(29) Conversely, high intakes of carotenoids and vitamin C, and high consumption of nutrient-dense vegetables and fruit are associated with lower levels of circulating hs-CRP,(30) and maintenance of normal body weight.(31, 32) Preferential intake of whole grains with lower glycemic load,(33) the elimination of trans fats, and provision of a favorable omega 3 to omega 6 ratio of polyunsaturated fats (PUFA) characteristic of the Mediterranean diet are also associated with attenuation of inflammation.(34) Essential PUFA need many co-factors such as folic acid, vitamin B12, vitamin B6, vitamin C, zinc, magnesium, calcium, L-arginine, and small amounts of selenium and vitamin E for full anti-atherosclerotic, anti-inflammatory activity.(35) This broad spectrum of nutrients has been demonstrated (in both animal and human studies) to be required for the immune system to function optimally.(23, 36) Innate, humoral, and mucosal immunity may be affected by deficiencies in one or more of these nutrients rendering an individual less able to modulate an appropriate immune response. The dynamic interface at the intestinal border, where the immune system is on constant guard, is therefore fundamentally connected to the nutrient quality of food. Vitamins A and D have specifically been shown to mediate intestinal epithelial defenses.(37-39) Enteral delivery of the amino acid glutamine, a substrate for the endogenous synthesis of arginine in most mammals, including humans, also contributes to gut-protective effects.(40) Recent evidence suggests glutamine's benefits are mediated via activation of peroxisome proliferator-activated receptor gamma,(41) a transcriptional pathway further discussed below.

"Non-nutritive" resistant and indigestible fibers are also critical for gut health and are deficient in the American diet.(42) Fiber intake is inversely proportional to hsCRP levels in NHANES analyses(43) and to insulin resistance in the Framingham offspring study.(44) with putative adverse consequences for weight regulation.(45) Resistant fibers, or prebiotics, are metabolized by gut flora to short chain fatty acids such as sodium butyrate, which nourish enterocytes and have favorable consequences for gut barrier function.(46) A theory being tested by our group, which is the basis for the bar having a high fiber matrix is that both the indigenous bacteria that depend on our dietary choices and the integrity of the gut wall are fundamentally important for host defense and systemic metabolic homeostasis. (Shigenaga, manuscript in preparation).

PRELIMINARY DATA- Stress, Inflammation, and the Metabolic Syndrome (SIMS)

We have accumulated substantial data that reflect the prevalence of nutrient deficiencies and insulin resistance in both obese and normal weight children at Children's Hospital and Research Center Oakland (CHRCO), 83% of whom are African-American (AA), Hispanic (H), or other minority races. (A.Lal, Mietus-Snyder, et al, manuscript in preparation) Presented are data on food intake based on the Block Kids' Food Frequency Questionnaire, and a brief summary of results of blood chemistry tests.

Food Intake: Among 31 obese children seen at CHRCO between 11-19 years of age with BMI ≥97th percentile, median intake of protein (193%), total fat (206%), saturated fat (246%), and carbohydrate (199%) were significantly greater than the RDA, as would be expected. However, the carbohydrate was predominantly refined and median fiber intake was only 46% of the RDA (less than the RDA in 81% of the subjects). The intake of essential omega3 and omega6 fatty acids, linolenic and linoleate, was inadequate in 42% and 45% of the sample respectively. The median intakes of magnesium, potassium, and calcium were strikingly low, consistent with the reported low average intake of fruits, vegetables (1.3 and 1.4 servings per day), and dairy products (0.8 serving per day). Between 81-93% of these obese children were consuming diets deficient in one or more of these key minerals, while 35% had low zinc intake. Significant vitamin deficiencies were also observed. Eighty-one per cent of children were deficient in vitamin E intake. Over one-third had suboptimal intake of at least one B vitamin, despite fortification in many processed foods, while close to half had diets low in vitamins C and K. Most children had suboptimal vitamin D intake, with the median intake being just 55% of RDA.

Blood Chemistry.: High fasting insulin (>17 IU), HOMA-IR (>2.5), and hsCRP (>1.0) were nearly universal, present in >90% of this obese pediatric cohort. Over 50% had elevated serum TG, and/or low serum HDL, with the caveat that the dyslipidemic manifestation of insulin resistance in AA children is evident at different thresholds (lower and higher respectively). Deficiencies of several vitamins were reflected in plasma levels. Low plasma alpha-tocopherol (≤5.5 mg/L) and vit C levels (≤0.5 mg/dL) were observed in 29% and 30% of the subjects respectively. Vit D levels were ≤80 nM in 74% and ≤50 nM in 63% of the subjects and the low serum inorganic phosphate (<3.6 mg/dL) present in 32% of the subjects may reflect physiologic effects of vit D deficiency. Hypovitaminosis D is more common in persons with darkly pigmented skin and is strongly associated with insulin resistance, inflammation, and beta cell dysfunction, which may be particularly important in this minority population. Since inflammation is strongly linked to poor iron utilization by the body, obesity could be a risk factor for iron deficiency. In our population of obese children, we discovered the prevalence of transferrin saturation <20% (iron depletion) was 73% and saturation <15% (iron deficiency) was 47%. Oxidized cysteine and glutathione, as well as homocysteine (Hcy) and cysteinyl glycine (Cys-Gly) levels were significantly elevated and total glutathione levels were reduced relative to normal weight children. These findings are consistent with increased oxidative stress in obese children that would be predicted to improve with the CB intervention, given our preliminary findings of decreased Hcy and Cys-Glc in healthy adults detailed below.

Dr. Mietus-Snyder has completed analyses of follow-up Block dietary data on an independent cohort of 28 obese, preadolescent inner city children (85% AA, H, or other minority, mean age 11.2 +/- 1.4, BMI 28.5 +/- 2.7) who participated in the 8 wk Stress, Inflammation, and the Metabolic Syndrome (SIMS) trial. All children completed the proposed group nutrition and activity counseling program and were randomized to either supervised exercise or mindfulness training. At baseline all children exhibit severe insulin resistance and inflammation and report low quality of life and heightened anxiety. Parent/guardians corroborate the poor quality of life, describe obesigenic child eating behaviors associated with eating without hunger, and report heighted levels of personal stress. The children were, as the above cohort, insulin resistant (HOMA 4.7 +/- 1.9, 90% > 3.0), dyslipidemic (TG 105 +/- 54, 25% > 130; HDL 39 +/- 11, 75% < 42), and inflamed (hsCRP 2.8 +/- 3.1, 75% > 1). Both groups achieved comparable weight stabilization with continued linear growth and improved, but still low self-report quality of life. Combined data revealed a trend towards reduction in BMI at 2 mo (28.0 +/- 2.5) which reached significance for BMI z-score (2.14 +/- 0.3 to 2.09 +/- 0.3, p = 0.02) but no measurable improvement in glucose homeostasis, or lipids. Baseline diets paralleled the above cohort: deficient in fiber, fruit, vegetable, dairy servings, and omega 3 fatty acids, high in saturated and trans fat, and deficient in magnesium, calcium, potassium and vitamins C, K, E, and D. There was no significant difference in diet by Block self-report in any of these nutritional parameters, though trends towards decreased total (35 +/- 5% daily kcal to 32 +/- 5, p = 0.08) and saturated fat intake (24 +/- 10 g/d to 19 +/- 5, p = 0.15) were noted. There was also a weak trend towards increased reported activity (p = 0.2). Despite weight stabilization after 2 mos of intensive counseling program therefore, barriers to lifestyle change continue to impede more robust progress towards dietary modification, weight loss, and improved metabolism.

We conclude that nutritional insufficiencies are prevalent in inner city obese children and that it is difficult for complex reasons to change dietary habits. Intensive family-based lifestyle counseling improves BMI z-scores and both child and parent quality of life at 2 months and our long term follow-up suggests that these benefits are sustained through one year of follow-up, but that diets remain poor, the quality of life suboptimal, and optimal metabolic health is not achieved. Concomitant improvement in insulin sensitivity may be impeded by the physiologic insulin resistance of puberty and/or by the persistently poor nutritional status of inner city children.

PRELIMINARY DATA- CHORIBAR

FORMULATION

The CB has been in development since August of 2005. CHORI metabolic researchers and nutrition scientists determined the types and quantities of nutritional supplements for the bar based on recent scientific research and the Dietary Reference Intakes and Upper Limits recommended by the Institute of Medicine, cognizant of average daily intake from other food and supplement sources from NHANES data, basic and clinical scientific evidence of bioactivity, and any known evidence of toxicity. Supraphysiologic doses of individual nutrients have yielded conflicting results in randomized trials, but there is solid evidence that a balanced diet confers benefit. When diet quality scores were calculated using the Nurses' Health Study comprehensive food frequency data and evaluated for their metabolic correlates, the Alternate Healthy Eating Index and Alternate Mediterranean Diet Score proved to be inversely associated with concentrations of biomarkers of inflammation and endothelial dysfunction.(47) The CB has been formulated to help achieve balanced nutrition with a polyphenolic-rich whole food matrix of with thirteen supplemental vitamins, choline, nine minerals, omega 3 fatty acids, a blend of insoluble and soluble fiber, protein, and glutamine at generally physiologic doses intended not to replace but to supplement a typical diet with the equivalent of the food pyramid's nutrient value

condensed in a bar. Exceptions added above 100% RDI in 2 bars are Vit D (as present RDI may be too low) and Vit C (as a preservative). Current cost is approximately $0.50 per bar. We have optimized flavor profiles and overcome production barriers presented by both nutrient instability and fat versus water solubility. The United States Department of Agriculture (USDA) Processed Foods Unit in Richmond, California, experienced in the manufacture, testing, quality-control, and packaging of nutritional supplements, is collaborating with CHORI by supplying a fruit base for the bar and preparing bar prototypes. Our USDA collaborators have a longstanding research interest in developing shelf-stable healthy foods from bulk-processed fruits and vegetables and their coproducts, facilitating adherence to MyPyramid® guidelines. All bars have extra natural blueberry flavoring, and stronger flavors have been added to mask the otherwise unfavorable taste associated with the bar's mineral and omega 3 fatty acid content. Either citric acid (sweet-sour) coating is added to the surface or decaf coffee beans are ground into the matrix – generating two bar flavors with comparable nutrient value, validated by Medallion® labs. The CB is nutrient-dense but moderate in calories (≈100 kcal in a 25 gm bar). To assure product safety at the moisture level deemed most palatable, microbiological analyses were conducted to rule out yeast, mold, total aerobic plate and coliform counts. The bars are packaged under nitrogen in Mylar foil laminate for optimum protection from oxidation of labile long chain fatty acids and antioxidant nutrients.

PILOT CHORIBAR STUDIES

This nutrient intervention has been piloted in three small short-term trials to test safety, acceptability, palatability, and short-term metabolic effects in adult volunteers who refrained from all other supplements.

The Phase I adult trial was conducted in 9 healthy adults with a focus on the palatability and safety of the bar taken twice daily for 2 wk. There were no reported gastroenterological symptoms despite the high fiber content of the bar, and the palatability was deemed acceptable. A significant rise in high density lipoprotein cholesterol (HDL) across a wide range of baseline values was observed (p=0.01). A Phase II trial was completed on 12 overweight and obese adults who ate the CB bid for one month, permitting us to further evaluate the metabolic impact of this form of nutrient supplementation. We again observed a significant increase in HDL cholesterol (p=0.02). Subtype analyses pooling data from these first two trials revealed that the elevation is specific to the large buoyant highly antiatherogenic type IIb subclass of HDL cholesterol (p=0.0001).

A trend towards elevated homocysteine levels was also noted in Phase 1, but the total values in this healthy cohort remained well within normal limits.

Phase II was conducted in an obese study population. Homocysteine (Hcy) levels again rose significantly in Phase II, but this obese study population had elevated baseline values, so the increase pushed some participants into clinically significant range.

Phase IIa: From the results of Phase II, we adapted the CB formulation for a Phase IIa study in 11 healthy adults with a modest increase in B vitamins (adjusting folate, Vit B1, B12, and B6 all to 50% RDA/bar). This proved sufficient to dissociate the HDL and Hcy changes. HDL still rose an average 5.2% (p = 0.018, range -5.7 to +/- 15.5%,) but this time we observed a decrease in Hcy (p= 0.004). As part of a redox metabolomics assay performed by Dr. Jung Suh in the Ames lab that evaluates cysteine metabolism, we also documented a significant drop in plasma cysteinyl-glycine (Cys-Gly) concentration (p=0.0001), a marker that has been associated with cardiovascular risk.(48) Reduction is consistent with improvement in metabolic health.

Total LDL has not changed significantly in any of our trials nor did LDL size change in participants for whom the major LDL species was type A or large and buoyant (>218.8 Å). The mean diameter for these subjects was 221.6 +/- 4.2 pre vs 221.6 +/- 5.2 post, p = 0.95) Subgroup analysis however reveals that in participants who began Phase IIa with a small dense type B (diameter < 215.5 Å) or intermediate (>215.5 but < 218.8 Å) LDL phenotype there is a trend towards increased LDL size.

Extended Phase IIa study: Ten short-term trials were conducted in adults to evaluate either hypothesized bioactive components of the bar or changes in the basic formulation. These trials are summarized in the table below and in Appendix 1:

Ph 2a extended study Part	Number of participants	CB formulation #	Formulation Change	Duration	Outcome
1	17	1	Fruit alone	2 wks	n/a
2	12	2	Fruit plus soluble fiber	2wks	n/a
3	11	3	Inclusion of B vitamins	2 wks	HDL elevated and Hcy reduced
4	26	4	Two forms of soluble fiber (beta glucan vs inulin)	2 wks	HDL elevated and Hcy reduced
5	16	5	soluble fiber (HPMC	2 months	new and favorable outcomes for insulin sensitivity but blunted the HDL and Hcy effects due to hypothesized excess barrier function of this highly viscous fiber.
6	8	6	full bar formulation with adjusted B vitamins	2 wks	HDL elevated and Hcy reduced
7	23	7	original formulation plus the addition of a new form of Vitamin K	2 months	principal outcomes of HDL elevation and Hcy reduction were confirmed but new palatability issues
8	10	8	fruit and soluble fiber	2 wks	bars without fruit or beta-glucan soluble fiber did not have the anticipated effect on HDL or Hcy
9	9	9	adds new components: lactate, resistant starch, freeze-fried blueberry	2 wks	favorable impact on texture and palatability; improvements in insulin sensitivity and LDL-cholesterol; comparable blunting of our HDL and Hcy efficacy
10		10	Same as #9	2 wks	In progress to re-test outcome of Part 9

Details of Ph2 extended short term trials:

Ph2a Part 1-n=17 (March 2008), the fruit component alone of the bars was tested for efficacy: none.

Ph2a-Part 2- n= 12 (April 2008), the fruit plus soluble fiber componets alone were tested for efficacy: none.

Ph2a-Part 3- n=11 (June 2008) -the full bar formulation with adjusted B vitamins as noted above following the Ph 2 finding of homocysteine elevation was tested with significant HDL elevation and Hcy reduction.

Ph2a-Part 4- n=26 (September 2008)- two alternate forms of soluble fiber (beta glucan vs inulin) were tested in a randomized study design that confirmed efficacy (HDL elevation and Hcy reduction) of the original formulation (with beta glucan) studied in Part 3.

Ph2a-Part 5- n= 16 (April through June 2009)- A longer - 2 month- trial of yet another form of soluble fiber (HPMC) gave new and favorable outcomes for insulin sensitivity but blunted the HDL and Hcy effects due to hypothesized excess barrier function of this highly viscous fiber.

Ph2a-Part 6- n=8 (September 2008)- A repeat trial of the original formulation tested in Part 3 (and one arm of Part 4) confirmed the reliability and reproducibility of the favorable HDL and Hcy effects.

Ph2a-Part7- n=23, with 3 dropouts due to palatability concerns (for the first time, Mar-May, 2010)- a 2 mo trial of the original formulation (used in parts 3, one arm of 4 and 6) plus the addition of a new form of Vitamin K. The principal outcomes of HDL elevation and Hcy reduction were confirmed but new palatability questions were raised that made us question the feasibility of testing of our current formulation in a high risk population from the Healthy Hearts clinic.

Ph2a-Part 8- n=10 (Feb 2010- note run out of sequence - ie just before part 7 due to the timing of bar production with the USDA- these bars were ready first- fewer required as this was a short- 2wk- and smaller trial)- this was a deconstruction trial designed to test the synergistic requirement of fruit and soluble fiber in the bars. Whereas the fruit and fiber have no effect alone (Ph2a-Part 2), bars studied in this small trial without fruit or beta-glucan soluble fiber did not have the anticipated effect on HDL or Hcy.

Ph2a-Part 9- n= 9 (Dec 2010)- This was a newly formulated bar that incorporates all of the key ingredients of our past successful trials and adds two new components felt to be important for optimal metabolism, lactate and a resistant starch, and importantly adds freeze-fried blueberry, a new form of fruit additive with unanimous participant agreement vis-à-vis the favorable impact on texture and palatability. We were encouraged to see improvements in insulin sensitivity and LDL-cholesterol in several participants in this trial- a finding we have not seen since Ph2a-Part 5 that tested HPMC- but interestingly we saw comparable blunting of our HDL and Hcy efficacy. This prompted the hypothesis that the addition of freeze-dried blueberry with its high pectin viscous, soluble fiber content, might have, like HPMC, provided both benefit and excessive barrier function. We are currently enrolling a repeat trial with this same bar in adult participants who have been in previous trials with half as much freeze dried blueberry to test this hypothesis.

It is of note that the main outcome of the bar, a significant increase in HDL, was only mildly attenuated in Ph2a-Part 9 (a comparable 5% increase in HDL was observed but at a borderline p value of 0.05, but there was still a favorable trend towards the large and buoyant HDL subspecies. Again, it is provocative

that in this same trial, several participants experienced improved insulin sensitivity and LDL reduction. These findings did not reach significance in the small, relatively insulin sensitive and normolipidemic cohort of 7 healthy adults but would be very positive added benefits to this nutrient intervention if replicated in the proposed high risk population.

Ph2a Part 10 We are currently reevaluating this near final formulation from Ph2a Part 9, a repeat of Part 9 with less freeze-dried blueberry.

The majority of participants in all trials have reported a significant satiating effect of the CB. When feedback from all three adult pilot trials of the current bar formulation are pooled, the mean drop in the hunger score on a 1 to 10 analog scale is 4.0 +/- 1.8 units 20 minutes after ingestion. None of these preliminary adult trials have been accompanied by weight loss, but neither have weight loss or specific nutrition and activity lifestyle guidelines been offered. The only instruction has been to eat a bar twice daily, with water. Many participants comment that the bars serve as a meal replacement. Therefore, despite the 200 kcal delivered with bid intake, there has been sufficient dietary adjustment to avoid any significant increase in weight after either the 2 week or 1 month trials. We have observed a suggestion of favorable weight redistribution: WC drops from a mean of 87.3 +/- 12.6 to 86.0 +/- 11.2 cm, p = 0.07.

Taste testing of the former formulation (deemed less palatable than the latest formulation we propose now to study) among 15 obese (BMI 47.8 +/- 19.5) preadolescents and teens (age 12.9 +/- 3.4) in the HH clinic was conducted in the summer of 2010 with the sweet/sour flavored bars. On an analog scale from 1 to 10, where 1 was unfavorable, 5 was acceptable, and 10 was favorable, the mean score was 5.9 +/- 3.1, (median 6). 67%, 73%, and 87% of participants could appreciate the fiber, fruit, and chocolate respectively. All but one said they would eat the bars if they knew they were good for them, including the 6 who scored the bar below 5 for taste. The single participant, who said he would rather not eat them if they were healthy, actually liked the bars, assigning a taste score of 8. We are encouraged by this generally positive reception to the nutrient-dense CB that our plan of studying an even more palatable version in a pediatric cohort who present to HH for weight management will be feasible. We are not sure however, that twice daily intake will be sustainable for adolescents beyond the first 2 wk or the study, as we have not yet tested the bar in a pediatric population. Therefore the study design of weekly group visits to closely monitor adherence for the 2 mo trial and the incorporation of parent/guardian participants for daily reinforcement was chosen.

11. Study Design: (Check all that apply).

☐ Placebo	☐ Blinded	☒ Randomized	☐ Investigational intervention without random assignment

If this study has any of the formal designations below, please indicate below: ☐ N/A

☐ Phase I	☒ Phase II	☐ Phase III	☐ Phase IV	☐ Open Label Extension

Additional description of <u>general study design</u>. Sequentially list all procedures, drugs or devices to be used on human subjects. Describe any use of placebos and indicate whether subjects will be randomized in this study. Attach flow diagram if appropriate. **If there are any investigational drugs, devices or biologic agents used in this study, please complete and attach the FDA Form 1572.** If this is an investigator-initiated study, attach the FDA Investigational Drug Application.

Study Population
**12. Characteristics of the Subject Population:** Describe the gender, ethnic background and health status. Provide a candid discussion of potential problems, if any, related to the study population. Explain the rationale for the use of special classes such as pregnant women, children, prisoners, wards of the court, or other vulnerable populations. If women, minorities, <u>non-English speaking subjects</u> or children are excluded, provide written justification.

Children and their parent/guardian will be recruited from the Oakland branch of the Children's Hospital Healthy Hearts (HH) preventive cardiology clinic.

The HH Clinic in Oakland sees an average of 12 new referrals per week, interspersed with follow-up visits over 2 ½ clinic days, serving a high proportion of disadvantaged inner city families of color; 24% African American, 58% Hispanic (predominantly Mexican or Latin-American), 6% Asian/Pacific Islander, 12% White. All of the children referred for weight management have a BMI > 85th percentile for age and gender (92% > 95th percentile).

Target enrollment will be 32 boys and girls and one parent guardian. The 32 boys and girls will be at > 95th percentile BMI who do not meet exclusion criteria. Families consent at the time of their HH intake visit to inclusion in the HH data base and opt in and out of the possibilty of contact for future research. Only families who have not opted out of this provision will be contacted.

We have had good adherence and follow-up in both HH clinic and with lifestyle counseling groups recruited from the HH clinic population, so are optimistic we will be able to identify families who are able to sustain this level of commitment. The plan to run the groups in the late afternoon or weekends should help minimize interference with adult work and child school schedules..

Because we have seen improvement in metabolic parameters with the CBs in persons along a wide spectrum of weight and presenting metabolic states, and because the 8 week group counseling sessions expand upon what is possible in one-on-one clinical visits, we do not feel that prior visits in the HH program preclude participation. On the contrary, prior visits as well as continued involvement in the HH clinical program after completion of the proposed study are deemed critical to reinforcement of lifestyle principles and long term success with weight management. Similarly there is no contraindication to parental participation if the parent is of normal weight. Most of the metabolic benefits documented to date in CB trials have been in healthy, normal weight adults.

13. Inclusion/Exclusion Criteria: Indicate the criteria for exclusion and inclusion and explain the system for equitable selection of subjects.

INCLUSION: Child:
- referral to HH clinic for weight management,
- age 14-18 yr,
- BMI > 95th percentile, and
- willingness to eat the CB bid after tasting a sample,
- Speaks English (as group sessions will be run in English),
- willingness to attend 8 weekly group counseling sessions.

INCLUSION: Parent/guardian:
- willingness to attend the 8 weekly sessions with their child.
- willingness to eat a CB

• report at the weekly visits

EXCLUSION: Child and parent/guardian:
• diabetes mellitus (49);
• hypertension (>95th percentile systolic or diastolic pressure);
• glucocorticoid therapy (> 15 mg/m2/d) (50) or
• inhaled steroid therapy for asthma (>1.5 mg/d or >0.75 mg/d for fluticasone propionate), (51) since adrenal suppression can occur that will affect inflammation and insulin sensitivity;
• weight loss drugs;
• lipid, or blood pressure lowering medication.

ADDITIONAL EXCLUSION: Parent/guardian:
• moderate or severe untreated hypertension but a parent can be on blood pressure lowering medication, if they have been on the dose stably for more than 3 mo preceding the study.
.

14. Eligibility for Study: How is eligibility determined and by whom?

Dr. Mietus-Snyder, study coordinator (to be named), Kirsten Laine-Graves, and HH physicians Drs. Tinajero-Deck and June Tester, will determine eligibility on the basis of review of the HH intake clinic record. All follow-up questioning by phone contact will be conducted by the research team, Drs. Mietus-Snyder, Kirsten Laine-Graves, and the study coordinator (to be named).

15. Duration of Subjects' Participation in the Study (include follow-up if applicable):

Once potential parent guardian/child dyads are identified in the HH data base, they will be contacted and if interested, scheduled for a visit in the PCRC for consent and screening. The goal is to identify 32 eligible dyads over one month. Interested families will have an opportunity to taste the bars at the time of informed consent. When all baseline assessments are completed the dyads will begin the 8 wk group therapy, receive weekly distribution of each successive week's supply of CBs and scheduled follow up visits. It is anticipated that a total of 4 months is required to complete the study.

Enrollment Plan and Recruitment

**16. Planned Enrollment at CHRCO:** Number of subjects needed to <u>complete</u> the study.	64 (32 adolescents & 32 adults)

**17. Enrollment plan:** If you expect failed screenings or subject withdrawals, will they be replaced until the appropriate numbers of subjects have completed the study? <u>If No, explain below</u>.	☐ Yes ☒ No ☐ N/A

We will make every effort to identify at the time of enrollment parent/child dyads who can commit to good study adherence and its completion. Given the 8 weekly group sessions that are built into this intervention, it would not be tenable to enroll additional children once the groups are underway. We are powered to see significant results in our main outcome, HDL cholesterol, with 12 per group so can accommodate up to 15% attrition in the control group (of 14 dyads) and 33% attrition in the intervention group (of 18 dyads). Opting for a slightly larger intervention group will both insure the adequacy of both adolescent and adult numbers for significance and may also increase the information available from our exploratory assays.

18. Recruitment: What methods will be used to identify and recruit potential subjects? Attach a copy of all planned advertisements, flyers and letters, etc. to potential subjects.

We will identify potential recruits within our HH research data base. The families in this data base have consented to be contacted for potential research studies. Interested families will be invited to attend an information session where they will learn about the study, have any questions answered and have an opportunity to taste the bars. If they are still interested in participating, the consent will be signed at this session and a baseline assessment will be scheduled on the PCRC.

Research Methods and Procedures

19. Methodology and Data Collection: Describe the research procedures that will be followed. Please list, in sequence, all study procedures, tests, and treatments required for the study. Please indicate those that are experimental and those that may be considered to be standard treatment. Include a detailed explanation of any experimental procedures. Attach table if available. Describe all activities involving human subjects and explain the frequency and duration of each activity.

Randomization: After all baseline bloodwork is assembled, adolescent-parent dyads will be dichotomized by HOMA-IR and randomized to represent comparable ranges of insulin resistance in two groups of 14 (control) and 18 (intervention).

Participants will be asked to consume a CHORI Bar twice a day with a minimum of 8 oz of water- once in the morning and once in the afternoon or evening.

Additionally, they will participate in 8 weekly group counseling session where they will learn about nutrition and activity and will have opportunity to participate in supervised exercise.

Participants will have three clinic visits: baseline, 2 weeks and 2 months. All procedures will be conducted at each visit.

MEASURES ON FASTING BLOOD SAMPLES (30 cc)

Inflammation is now an established risk factor for many chronic diseases, including the metabolic syndrome. Inflammation affects insulin sensitivity and vascular function in a dynamic and highly interdependent fashion involving cross talk with markers of vascular oxidative stress and dyslipidemia. Multiple well validated indices of inflammation and insulin resistance will be monitored in parallel with an objective assessment by questionnaire of family health history, physical activity, dietary intake and micronutrient status. We will measure blood pressure, height, weight, and waist circumference and will obtain fasting bloodwork for serum glucose, insulin, lipoprotein profile, liver function tests, blood urea nitrogen and creatinine, complete blood count, high sensitivity C-reactive protein, ghrelin, folate, and vitamins B12, C, D, E, and iron. The fasting glucose and insulin will be used to calculate the homeostatic model of insulin resistance (HOMA-IR = Fasting glucose [mmol/L] X fasting insulin [mU/L]/ 22.5).50 In addition to these biomarkers: new and promising analytic techniques are being developed in the Ames and Shigenaga Laboratories at CHORI that may shed new light on the etiology and optimal management of the MetS.

Physical Measures
Measurement of subcutaneous vs. visceral fat
We refer to “visceral fat” throughout this proposal, but we recognize that anthropometric measures may not distinguish as accurately between visceral and subcutaneous fat as imaging methods, yet are more readily and economically attained. To get a proxy measure of visceral fat, we will measure waist

circumference half way between the lower costal margin and the iliac crest.

Blood pressure monitoring
In so much as hypertension is one of the principle findings of the metabolic syndrome, careful blood pressure monitoring will be key to the assessment of CV risk in the study cohorts. A Dynamap oscillometric device will be used to measure systolic, diastolic, and mean arterial pressure, with repeat measures taken by auscultation. We will follow established guidelines for BP measurement in the right arm, using a cuff of the appropriate size with the arm at heart level with the participant seated.

Lab outcomes relating to insulin, dyslipidemia, inflammation, and satiety
A single blood draw of 20 mL volume divided between EDTA and heparin tubes will be used to perform the laboratory analyses in the Children's Hospital Oakland research and clinical laboratories, or CLIA-certified reference lab, as required. An additional 7 ml of whole blood will be drawn for the performance of new experimental assays, to be processed as detailed below, under the description of these tests.

Fasting insulin and glucose to assess insulin homeostasis
Blood samples will be collected from the antecubital vein of recumbent subjects for insulin and glucose. Insulin will be analyzed using commercially available double antibody radioimmunoassay kits purchased from Linco Research (St. Charles, MO). Glucose will be measured using a YSI glucose analyzer Model 2300 (Yellow Springs Instruments, Yellow Springs, OH). If there is not adequate staffing to run the insulin and glucose measures in house, they will be sent out to ARUP laboratories (Salt Lake City, UT). The HOMA and QUICKI will both be calculated from the fasting glucose and insulin values.(52)

Fasting lipoprotein profile and sizing (Krauss laboratory, CHORI)
A simple screen of TC, TG, and HDL using heparin manganese polyanion precipitation obviates the need for ultracentrigugation and permits accurate calculation of LDL via the Friedwald equation, assuming fasting TG levels below 400 mg/dl. Focus on TG, HDL, and on the TG/HDL ratio provides by inference considerable information about hepatic insulin resistance. (53) The mixed dyslipidemia of the metabolic syndrome, characterized by hyperTG and low HDL, is influenced in the insulin resistant state by a dynamic reciprocal, coordinated decrease in lipoprotein lipase (LPL) and increase in hormone-sensitive lipase (HSL) enzymatic activity.(54) While total LDL cholesterol may be unremarkable, the LDL phenotype is typically small, dense, and atherogenic,(55) tracks with hsCRP(56) (indicative of the proinflammatory state of insulin resistance).

High-sensitivity C-reactive protein (hs-CRP) (ARUP laboratories, SLC, UT)
CRP is a hepatic acute phase protein largely regulated by circulating levels of IL-6. CRP increases rapidly in response to inflammation and decreases just as rapidly with the resolution of the condition. Chronic low level CRP elevation is characteristic of obesity and the MetS, is proatherogenic, and can be identified with a high sensitivity assay.(57)

Nutrient Assessment (Vits C & E, Ames lab; Vit D & iron, ARUP laboratories, SLC, UT)
Plasma Vitamin and Mineral levels - C, D, E, and iron will be measured in the plasma.

Measurements of food intake and activity, palatability, hunger and satiety
Block food frequency (FFQ) and Physical Activity (PA) questionnaires
At each assessment visits, macronutrient intake will be quantified using the Block FFQ, which is validated in overweight and normal-weight adults and adolescents of multiple ethnic backgrounds, and is available in Spanish.(58) The PA questionnaire is a brief assessment of standard activity.
A visual analog scale standardly used for assessment of hunger and satiety preceding and following meals will be used in effort to capture general feelings of hunger and satiety from participants.

Tests of Fitness and Metabolism: The fitness 'step test' involves stepping up and down on a single step at a steady pace for 3 minutes and monitoring pulse taken at the start and finish. For the breathing test, participants will be asked to lie down quietly under a clear respirometer mask that will measure the air breathe in and out for several minutes at rest to calculate a resting energy quotient, a sensitive indicator of mitochondrial function.

Optional plasma storage and DNA testing: If participants consent, the plasma and cells that would otherwise have been discarded will be saved for potential future research questions. A buffy coat isolation to prepare genomic DNA for telomere and mitochondrial DNA assessment. We will also collect buccal samples for a second mitochondrial tissue analysis from participants who consent for DNA studies. These studies are evaluating the stability of DNA related to nutrient status but are not characterizing genomic DNA sequence.

20. Surveys, questionnaires, or psychological tests: If applicable, please describe the provisions for administering these measures, the mode of administration, the setting, and if special training or qualifications are necessary. ☐ N/A

Standard validated quality of life instruments will be performed at baseline and 2 mo follow-up, including:
1. Adult and Adolescent MacArthur Scales of Subjective Social Status
2. Perceived Stress Inventory (parent and adolescent report)
3. Pediatric Quality of Life Inventory (parent and adolescent forms)
4. Block Food Frequency Questionnaire and Activity Questionnaire (adult and adolescent)
5. Participant Feedback (palatability, timing of ingestion, satiety) questionnaire (same version for both adult and adolescent participants)

21. Data Storage: Please complete the following questions regarding data storage:

a. How will the data be collected and recorded? How will the data be coded to protect personal privacy?

Questionnaire data will be collected on paper and entered into a HIPAA compliant and password protected online data base.
Laboratory samples will be processed by the PCRC lab personnel for deidentified sendout to ARUP CLIA certified labs or sent diedentified to CHORI investigators for research analysis. Dr. Mietus-Snyder with assistance from study coordinator will maintain a confidential, password protected data base on the Ames laboratory secure server.

b. How will the data be stored during the study?

The data will be stored in the online data base or on the CHORI Ames lab server.

c. Who will have access to the data and the data codes? If data with subject identifiers will be released, specify the person(s) and agencies to whom this information will be released?

The PI and study team will have password protected access to the study data.

d. What will happen to the data when the study is completed?

All data will be converted to an Excel spread sheet and coupled with the ARUP and CHORI lab data,

then closed/locked to further manipulation. All data files for long term storage will be identified by the participant's study ID only. The PI will maintain the study decoder in a separate password protected, locked file in the event that any study participant requests that any extra plasma stored (by consent) long term be destroyed.
**Risk and Benefit Assessment**
**22. Potential Risks and Discomforts:** Describe any potential risks or likely adverse effects of the drugs, biologics, devices or procedures subjects may encounter in the study. State the potential risks – physical, psychological, social, legal or other – connected with the proposed procedures and assess their likelihood and seriousness.
There is a small risk of pain, bruising, or infection with venipuncture. There are possible psychological risks of completing questionnaires, particularly those aimed at identifying the participant's eating and activity habits. The acquisition of physical measures, especially weight and abdominal circumference, may make some participants uncomfortable. Participation in research may involve a loss of privacy, but all health information will be handled as confidentially as possible with substitution of study ID numbers in place of names on all study forms, questionnaires, and blood tubes.
**23. Safety Precautions for Minimizing Risks:** Describe the procedures for minimizing any potential risks. Where appropriate, discuss provisions for ensuring necessary medical or professional intervention in the event of adverse effects to the subject.
The small risk of venipuncture will be minimized by using trained phlebotomists or experienced MD's and strict sterile technique. The acquisition of all physical measures will be done with care to protect privacy . Participation in research may still involve some loss of privacy, but all health information will be handled as confidentially as possible with substitution of study ID numbers in place of names on all study forms, questionnaires, and blood tubes.
**24. Benefit Ratio:** What is the risk benefit ratio of this research, compared with available alternatives? Describe the potential benefits the subjects may receive as a result of their participation in the research and what benefits to society may be expected. **For greater than minimal risk research involving children there must be the prospect of direct benefit to the individual subjects.**<br>Note: The potential benefits of the research must justify the risks to human subjects. The risk benefit ratio of the research must be at least as favorable for the subjects as that presented by standard treatments for their condition. When comparing the risk/benefit ratio of research with that of available alternatives, the alternative of doing nothing should be included in the analysis.
Given the minimal risk associated with this study, the potential benefits of receiving personal health information and of potentially improving one's nutritional status should outweigh any risk. The 8 weekly visits will be an imposition on the particpants' time, but the provision of intensive weekly lifestyle counseling may provide lifetime tools of value for maintaining a healthy weight. It may prove difficult to eat the CBs twice daily for a full 2 months, but many participants in past trials have reported that the provision of this healthful snack has helped them feel better. The alternative to participation is simply to not participate. There will be no adverse effects on ongoing care in the HH clinic to any potential participants.
**25. Therapeutic Alternatives:** What therapeutic alternative(s) are reasonably available to potential subjects should they choose not to participate in the study? These may be research or non-research based alternatives.
There are no alternatives to this trial.

Financial Considerations
**26. Payment for Participation:** Describe all plans to compensate subjects, including provision of services, and other reimbursements. Describe the conditions that subjects must fulfill to receive full or partial pro-rated payment.
Each participant dyad receives a $10 incentive at orientation and each weekly counselling visit ($90), a $20 incentive at the baseline, 2 wk, and 2 mo PCRC physical assessment visits ($60), plus a $50 gift card at the final PCRC visit at the time of study completion or a total of $200. Weekly sessions will take place at CHORI where parking is free. Parking vouchers will be provided for the baseline and 2 mo visits. All of the testing will be paid for by the research study.
**27. Financial Obligations of Subjects:** Will subjects have to pay for any of the tests or treatments that they receive as part of the research? Please clarify who will pay for the procedures associated with the study as well as procedures that may be part of standard clinical care. Clarify that insurance and other third party payers may not cover standard procedures if they are associated with a research project.
There is no financial obligation for participants
**28. Emergency Care and Compensation for Research-Related Injury:** If the research presents an unknown or greater than minimal risk illness/injury, the financial liability for the costs of care associated with the potential research related illness/injury must be specified. If no funds are available, please include language to explain this to potential subjects.
This research does not present an unknown or greater than minimal risk of illness/injury. There are no funds for financial liability for unknown risks.
**Informed Consent**
**29. Capacity to Consent:** Will all subjects have the capacity to give informed consent? If not, describe the likely range of impairment and explain how, and by whom, their capacity to consent will be determined.
Adult participants will have full capacity to consent for themselves and for their adolescent children. Adolescent participants will have capacity to assent.
**30. Study Personnel Administering the Consent Process:** Please identify by name and training the individual(s) who will be authorized to describe the research to subjects or their representatives, and to invite their participation. To insure that subjects give complete informed consent and are able to ask and have answered all questions regarding the nature of their participation, the personnel administering the consent must have appropriate training and background.
Drs. Michele Mietus-Snyder, the study coordinator (to be named), Kirsten Laine-Graves will take responsibility for obtaining the study consents and assents.

31. Process of Consent: Please discuss how the consent process will be conducted, describing the following elements:

a) The environment and location where the informed consent will be solicited;
b) Opportunities for the potential subjects to discuss their participation with family or others before signing the consent form;
c) How and by whom it will be determined whether the subject or their legally authorized representatives understand the information provided; and
d) **The types of forms used** (e.g., adult consent form, parental permission form, combined form, translations to other languages, etc.)

a) Healthy Hearts (HH) clinic and/or the PCRC will be the sites for consent
b) The study requirements, risks, and benefits, and the complete voluntary nature of participation will be reviewed
c) Drs. Mietus-Snyder, Kirsten Laine-Graves, and the study coordinator to be named) will obtain the informed consent and confirm study requirments are understood.
d) adult consents and child assents

32. Assent of Minor: For subjects age 7 through 17: ☐ N/A (under 7 or adult)

a) Considering the subject's potential capacity and medical condition, what is the suggested age of the minors to provide assent?	14-17

b) Detail below whether the assent should be in writing (a separate assent form signed by the child), and/or obtained orally.

The separate assent will be in writing for signature by the child.

c) Would it would be appropriate to include on the consent form a signature block for adolescents who are able to understand the adult consent form **(minimal risk studies only).**	☐ Yes<br>☒ No

d) Detail any justification for requesting that the IRB waive assent. ☒ N/A

33. Information Withheld from Subjects: If any information about the research purpose and design of the study will be withheld from subjects, please explain the non-disclosure and describe plans for post-study de-briefing. ☒ N/A

No information will be withheld

Data Analysis

34. Statistical Analysis: Please delineate the data analysis plans for this study. Include planned statistical analyses and explanation of determination of sample size. Briefly describe what statistical analysis(es) of which outcome will be applied to address each primary aim. Examples of statistical analyses include:

Calculation of descriptive statistics such as mean, median, SD, range, tallies.
Examination of graphs such as outcome vs. time, scatterplots of two variables, Kaplan-Meier curves.
Estimation of differences between two groups with comparison by t-test or Mann-Whitney test.

Estimation and testing of within-person changes by matched t-test or Wilcoxon signed-rank test.
Multiple linear regression, logistic regression, or Cox proportional hazards regression.
Repeated measures models (usually requires the help of a statistician).

For qualitative research, briefly describe how qualitative data will be analyzed.

We will evaluate the relationship(s) between micronutrient status and metabolic homeostasis at baseline in obese inner city children and their parents, and the additive impact of the CB nutritional intervention on metabolism and weight loss in children and their parents enrolled in an intensive lifestyle intervention. We are fortunate to have considerable pilot data for what is still exploratory research as we confirm physiologic mechanisms for the observed outcomes. We have powered our sample size based upon reproducible changes in HDL, Hcy, and Csy-Gly levels that have been observed in paired t-test analyses in our short-term trials. Extrapolating from the cumulative observed effects on HDL (5.4 +/- 4.7% increase) as well as on the large HDL subspecies HDL2b (424 +/- 367 nmol/L), we have 80% power to detect a difference in preadolescent and adult participants in all of these key outcomes, with a two-sided alpha of 0.05 if we enroll 12 children in each arm. Because this is a randomized trial, we will enroll additional participants in two groups slightly weighted towards the intervention (14 control and 18 intervention dyads) to allow for attrition while still protecting statistical power to see significant results in both pre-post comparisons within groups and between control and intervention groups for both adolescents and children. The overall effect for Hcy reduction of 2.4 +/- 3.6 ucml/L would require a sample size of 35 per group which we cannot afford to enroll at this time. Since we have observed in past adult trials a trend towards greater Hcy reduction at higher baseline values it is possible that an even larger effect size in Hcy will emerge from the cohort of obese preadolescent participants at increased risk for hyperhomocysteinemia. If so, our target sample size may also be adequate to also assess Hcy change.

Secondary outcomes include LDL and HDL diameter change, hs-CRP, HOMA-IR, fasting TG, BMI (and BMI z-score) and visceral adiposity (assessed by both waist circumference and abdominal height), blood pressure, and psychosocial measures.

Our comparison study design will permit both between group unpaired analyses and within group paired longitudinal analyses. The null hypothesis is that there is no association between observed physical, metabolic outcomes and predictor variables at either baseline or following the intervention. For cross sectional analyses, our primary predictor variables will derive from nutrient analyses (Block FFQ data), focused on baseline fatty acid, polyphenolic, and fiber intake.

For longitudinal analyses, our primary predictor variable will be CB intake. We will ask participants to report intake at weekly intervals when additional bars will be distributed. The known nutrient value of the CB intervention will be factored into the Block analyses, and dietary change will be assessed. We know that plasma levels of vitamins C, D, and E are low in obese inner city children but do not know if these values will change with lifestyle counseling alone or with counseling plus the CB. Self report of bar intake has proven sufficient in our pilot studies for assessment of compliance, but we will explore plasma vitamin levels as more objective measures of compliance that have been used successfully in outpatient nutrition research.

With the exception of a single one month trial in overweight adults that preceded the final CB formulation (with B vitamin adjustments) and a recent 2 mo trial of the slightly different formulation in healthy adults, all of our CB pilot data have been generated after a two wk intervention. The extension of the first pediatric trial for two months in the context of weight loss counseling allows us to both evaluate the sustainability of any observed 2 wk outcomes and to more fully explore endpoints we have

not yet observed with the CB. These include weight loss, lowered hsCRP, and insulin sensitivity as reflected by change in HOMA-IR.

Comments/Remarks

This area may be used as continuation of other items. You may also attach additional sheets as necessary.

We recognize that adherence to the bid intake of the CB intervention in the outpatient setting is a limitation, but it is also necessary to overcome if any benefits observed can be generalized. Our proposed study design permits us to see participants weekly for the duration of the 2 mo intervention and to mobilize parental support by engaging them in the full intervention and follow-up. We will maintain regular telephone, and if possible e-contact, depending upon the participant's access to a cell phone and/or computer. There are only modest financial incentives as funding is limited, but they amount to $200 per family dyad by the end of the study. We have seen excellent (>95%) adult compliance with bar intake to date without incentives. In pilot tasting sessions with overweight preadolescents and adolescents from our target population, sensory feedback was positive. Children who struggle with their weight have volunteered that they are interested in anything that can help them manage the hunger that they perceive controls so many lifestyle decisions, and the bars are universally reported to promote satiety. Futhermore, the majority of preadolescents in our sensory testing pilot reported they would eat these bars twice daily if they felt it would benefit their health. Given the reliability and reproducibility of positive key outcomes in repeated adult pilot studies across a wide range of ages and baseline physiology, we anticipate favorable metabolic changes will also be observed in the proposed pediatric cohort.

Attachments

Please list <u>Attachments, Supplements and Appendices</u>, including Version(s) and date(s).

BIBLIOGRAPHY

1. Zhang L, Bruce-Keller AJ, Dasuri K, Nguyen AT, Liu Y, Keller JN. Diet-induced metabolic disturbances as modulators of brain homeostasis. Biochim Biophys Acta 2009; 1792: 417-422.

2. Zaalberg A, Nijman H, Bulten E, Stroosma L, van der Staak C. Effects of nutritional supplements on aggression, rule-breaking, and psychopathology among young adult prisoners. Aggress Behav 2010; 36: 117-126.

3. Bachman JL, Reedy J, Subar AF, Krebs-Smith SM. Sources of food group intakes among the US population, 2001-2002. J Am Diet Assoc 2008; 108: 804-814.

4. Thorson E, Beaudoin CE. The impact of a health campaign on health social capital. J Health Commun 2004; 9: 167-194.

5. Howard BV, Van Horn L, Hsia J, Manson JE, Stefanick ML, Wassertheil-Smoller S, et al. Low-fat dietary pattern and risk of cardiovascular disease: the Women's Health Initiative Randomized Controlled Dietary Modification Trial. Jama 2006; 295: 655-666.

6. MacIntyre S, Ellaway A, Cummins Sp-. Place effects on health: how can we conceptualise, operationalise and measure them? Social Science & Medicine 2002; 55: 125-139.

7. Dubowitz T, Heron M, Bird CE, Lurie N, Finch BK, Basurto-Davila R, et al. Neighborhood socioeconomic status and fruit and vegetable intake among whites, blacks, and Mexican Americans in the United States. Am J Clin Nutr 2008; 87: 1883-1891.

8. Guthrie JF, Lin BH, Frazao E. Role of food prepared away from home in the American diet, 1977-78 versus 1994-96: changes and consequences. J Nutr Educ Behav 2002; 34: 140-150.

9. Laugero KD. Filling in the gaps of chronic psychological stress disease models: what's metabolic profiling have to do with it? Endocrinology 2008; 149: 2712-2713.

10. Snijder M, vanDam R, Visser M, Deeg D, Dekker J, Bouter L, et al. Adiposity in relation to vitamin D status and parathyroid hormone levels: a population-based study in older men and women. J Clin Endocrinol Metab 2005; 90: 4119-4123.

11. Parikh S, Edelman M, Uwaifo G, Freedman R, Semega-Janneh M, Reynolds J, et al. The relationship between obesity and serum 1,25-dihydroxy vitamin D concentrations in healthy adults. J Clin Endocrinol Metab 2004; 89: 1196-1199.

12. Molnar D, Decsi T, Koletzko B. Reduced antioxidant status in obese children with multimetabolic syndrome. Int J Obes Relat Metab Disord 2004; 10: 1197-1202.

13. Reitman A, Friedrich I, Ben-Amotz A. Low plasma antioxidants and normal plasma B vitamins and homocysteine in patients with severe obesity. Isr Med Assoc J 2002; 4: 590-593.

14. Wallstrom P, Wirfalt E, Lahmann P, Gulberg B, Janzon L, Berglund G. Serum concentrations of beta-carotene and alpha-tocpheral are associated with diet, smoking, and general and central adiposity. Am J Clin Nutr 2001; 73: 777-785.

15. Neuhouser M, Rock C, Eldridge A, Kristal A, Patterson R, Cooper D, et al. Serum concentrations of retinol, alpha-tocopherol and the carotenoids are influenced by diet, race and obesity in a sample of healthy adolescents. J Nutrition 2001; 131: 2184-2191.

16. Strauss R. Comparison of serum concerntrations of alpha-tocopherol and beta-carotene in a cross-sectional sample of obese and nonobese children (NHANES III). Ntional Health and Nutriiton Examination Survey. J Pediatr 1999; 134: 160-165.

17. Moor de Burgos A, Wartanowicz M, Ziemlanski S. Blood vitamin and lipid levels in overweight and obese women. Eur J Clin Nutr 1992; 46: 803-808.

18. Ames B. A role for supplements in optimizing health: the metabolic tune-up. Arch Biochem Biophys 2004; 423: 227-234.

19. Liu S, Willett WC, Manson JE, Hu FB, Rosner B, Colditz G. Relation between changes in intakes of dietary fiber and grain products and changes in weight and development of obesity among middle-aged women. Am J Clin Nutr 2003; 78: 920-927.

20. Patrini C, Griziotti A, Ricciardi L. Obese individuals as thiamin storers. Int J Obes Relat Metab Disord 2004; 28: 920-924.

21. Olusi S, Al-Awadhi A, Abiaka C, Abraham M, George S. Serum copper levels and not zinc are positively associated with serum leptin concentrations in the healthy adult population. Biol Trace Elem Res 2003; 91: 137-144.

22. Johnston C. Strategies for healthy weight loss: from vitamin C to the glycemic response. J Am Coll Nutr 2005; 24: 158-165.

23. Calder PC, Kew S. The immune system: a target for functional foods? Br J Nutr 2002; 88 Suppl 2: S165-177.

24. Basu A, Devaraj S, Jialal I. Dietary factors that promote or retard inflammation. Arterioscler Thromb Vasc Biol 2006; 26: 995-1001.

25. Engstrom G, Hedblad B, Stavenow L, Lind P, Janzon L, Lindgarde F. Inflammation-sensitive plasma proteins are associated with future weight gain. Diabetes 2003; 52: 2097-2101.

26. Thurnham DI, Mburu AS, Mwaniki DL, De Wagt A. Micronutrients in childhood and the influence of subclinical inflammation. Proc Nutr Soc 2005; 64: 502-509.

27. Schenkel TC, Stockman NK, Brown JN, Duncan AM. Evaluation of energy, nutrient and dietary fiber intakes of adolescent males. J Am Coll Nutr 2007; 26: 264-271.

28. Feldeisen SE, Tucker KL. Nutritional strategies in the prevention and treatment of metabolic syndrome. Appl Physiol Nutr Metab 2007; 32: 46-60.

29. Zhang X, Zhang G, Zhang H, Karin M, Bai H, Cal D. Hypothalamic IKKb/NFkB and ER stress link overnutrition to energy imbalance and obesity. Cell 2008; 135: 61-73.

30. Nanri A, Moore MA, Kono S. Impact of C-reactive protein on disease risk and its relation to dietary factors. Asian Pac J Cancer Prev 2007; 8: 167-177.

31. Sarter B, Campbell TC, Fuhrman J. Effect of a high nutrient density diet on long-term weight loss: a retrospective chart review. Altern Ther Health Med 2008; 14: 48-53.

32. Savage JS, Marini M, Birch LL. Dietary energy density predicts women's weight change over 6 y. Am J Clin Nutr 2008; 88: 677-684.

33. Qi L, Hu FB. Dietary glycemic load, whole grains, and systemic inflammation in diabetes: the epidemiological evidence. Curr Opin Lipidol 2007; 18: 3-8.

34. Giugliano D, Esposito K. Mediterranean diet and metabolic diseases. Curr Opin Lipidol 2008; 19: 63-68.

35. Das UN. Essential fatty acids: biochemistry, physiology and pathology. Biotechnol J 2006; 1: 420-439.

36. Zhao G, Etherton TD, Martin KR, Vanden Heuvel JP, Gillies PJ, West SG, et al. Anti-inflammatory effects of polyunsaturated fatty acids in THP-1 cells. Biochem Biophys Res Commun 2005; 336: 909-917.

37. McCullough FS, Northrop-Clewes CA, Thurnham DI. The effect of vitamin A on epithelial integrity. Proc Nutr Soc 1999; 58: 289-293.

38. Zasloff M. Fighting infections with vitamin D. Nat Med 2006; 12: 388-390.

39. Kong J, Zhang Z, Musch MW, Ning G, Sun J, Hart J, et al. Novel role of the vitamin D receptor in maintaining the integrity of the intestinal mucosal barrier. Am J Physiol Gastrointest Liver Physiol 2008; 294: G208-216.

40. Wang J, Chen L, Li P, Li X, Zhou H, Wang F, et al. Gene Expression Is Altered in Piglet Small Intestine by Weaning and Dietary Glutamine Supplementation. J Nutrition 2008; 138: 1025-1032.

41. Ban K, Kozar RA. Enteral glutamine: a novel mediator of PPARgamma in the postischemic gut. J Leukoc Biol 2008; 84: 595-599.

42. Leach JD. Evolutionary perspective on dietary intake of fibre and colorectal cancer. Eur J Clin Nutr 2007; 61: 140-142.

43. Ajani UA, Ford ES, Mokdad AH. Dietary fiber and C-reactive protein: findings from national health and nutrition examination survey data. J Nutr 2004; 134: 1181-1185.

44. McKeown NM, Meigs JB, Liu S, Saltzman E, Wilson PW, Jacques PF. Carbohydrate nutrition, insulin resistance, and the prevalence of the metabolic syndrome in the Framingham Offspring Cohort. Diabetes Care 2004; 27: 538-546.

45. Pereira MA, Ludwig DS. Dietary fiber and body-weight regulation. Observations and mechanisms. Pediatr Clin North Am 2001; 48: 969-980.

46. Brouns F, Kettlitz. B, Arrigoni E. Resistant starch and "the butyrate revolution" Trends in Food Science & Technology 2002; 13: 251-261

47. Fung TT, McCullough ML, Newby PK, Manson JE, Meigs JB, Rifai N, et al. Diet-quality scores and plasma concentrations of markers of inflammation and endothelial dysfunction. Am J Clin Nutr 2005; 82: 163-173.

48. Giral P, Jacob N, Dourmap C, Hansel B, Carrie A, Bruckert E, et al. Elevated gamma-glutamyltransferase activity and perturbed thiol profile are associated with features of metabolic syndrome. Arterioscler Thromb Vasc Biol 2008; 28: 587-593.

49. Report of the expert committee on the diagnosis and classification of diabetes mellitus. Diabetes Care 1997; 20: 1183-1197.

50. Karssen A, Meijer O, Berry A, Sanjuan P, de Kloet E. Low doses of dexamethasone can produce a hypocoticosteroid state in the brain. Endocrinology 2005; 146: 5587-5595.

51. Lipworth B. Systemic adverse effects of inhaled corticosteroid therapy: A systmeatic review and meta-analysis. Arch Intern Med 1999; 159: 941-955.

52. Keskin M, Kurtoglu S, Kendirci M, Atabek M, Yazici C. Homeostasis model assessment is more

reliable than the fasting glucose/insulin ratio and quantitative insulin sensitivity check index for assessing insulin resistance among obese children and adolescents. Pediatrics 2005; 115: e500-503.

53. Kohen-Avramoglu R, Theriault A, Adeli K. Emergence of the metabolic syndrome in childhood: an epidemiological overview and mechanistic link to dyslipidemia. Clin Biochem 2003; 36: 413-420.

54. Frayn K, Coppack S, Fielding B, Humphreys S. Coordinated regulation of hormone-sensitive lipase and lipoprotein lipase in human adipose tissue in vivo: implications for the control of fat storage and fat mobilization. Adv Enzyme Regul 1995; 35: 163-178.

55. Tribble D, Rizzo M, Chait A, Lewis D, Blanche P, Krauss R. Enhanced oxidative susceptibility and reduced antioxidant content of metabolic precursors of small, dense low-density lipoproteins. American Journal of Medicine 2001; 110: 103-110.

56. St-Pierre A, Bergeron J, Pirro M, Cantin B, Dagenais G, Despres J, et al. Effect of plasma C-reactive protein levels in modulating the risk of coronary heart disease associated with small, dense, low-density lipoproteins in men. Am J Cardiol 2003; 91: 555-558.

57. Rifai N, Tracy R, Ridker P. Clinical efficacy of an automated high-sensitivity C-reactive protein assay. Clinical Chemistry 1999; 45: 2136-2141.

58. Block G, Subar A. Estimates of nutrient intake from a food frequency questionnaire: the 1987 National Health Interview Survey. Journal of the American Dietetic Association 1992; 92: 969-977.

CHILDREN'S HOSPITAL
& RESEARCH CENTER OAKLAND

Study Abstract (For CHORI Website)
Provide an abstract/full synopsis of this study to be posted on the CHORI Website.<br>http://www.chori.org/Human_Research/Human_Research/active_studies_home.html<br><br>**ABSTRACT**<br>The prevalence of pediatric obesity in the United States has increased during the past three decades for complex reasons, which are likely to include increased consumption of refined diets rich in calories but deficient in fiber and micronutrients. The resultant micronutrient malnutrition has been argued by our group and many others to contribute to inflammation and metabolic dysregulation, which augment risk for cardiovascular and other chronic diseases. Minority populations are disproportionately affected by both obesity and its metabolic comorbidities, and have limited access to proven lifestyle behaviors that can prevent these conditions, including access to healthy food. We have developed the CHORIBAR (CB) as an economical nutritional supplement that provides a source of micronutrients and fiber largely inadequate in the typical American diet. Preliminary data suggest that twice daily intake of the CB for two weeks raises high density lipoprotein cholesterol (HDL) and favorably increases HDL size. These improvements in the dyslipidemia commonly associated with obesity suggest that the CB is restoring healthier metabolism, which is consistent with parallel observations of lowered homocysteine and a trend toward more favorable weight redistribution. Balanced nutrition may be required to attenuate inflammation, catalyze key enzymes, enhance mitochondrial biogenesis and metabolic homeostasis. Our hypothesis proposes that a 2 mo trial of CB intake in obese inner city, largely minority, adolescent children will improve nutritional status, lipid metabolism, glucose and energy homeostasis, hunger/satiety, body weight and adipose distribution. We further hypothesize that adherence to this nutritional intervention will be improved by enrolling adolescents together with a participating parent/guardian. Obese minority children have a high prevalence of metabolic dysregulation and an effective, inexpensive nutritional intervention that attenuates the subclinical inflammation which underpins numerous chronic disease processes, would have substantial public health ramifications.
