# Supplementary material for: Randomized nutrient bar supplementation improves exercise-associated changes in plasma metabolome in adolescents and adult family members at cardiometabolic risk
Source: PLoS One. 2020 Oct 20;15(10):e0240437. doi: 10.1371/journal.pone.0240437 (PMC7575082; doi:10.1371/journal.pone.0240437)
Supplement: S1 Flowchart — (DOCX) [file pone.0240437.s002.docx]

| **Stage** | **Number of people included** | **Number of people not included or excluded** | **Rationale** |
| --- | --- | --- | --- |
| Enrolment | 41 family units evaluated for potential enrolment | 23 units did not meet the inclusion criteria or met the inclusion criteria but declined to be enrolled after tasting the intervention nutrient bar | The participants enrolled are representative of the demographic and metabolic distribution common to the weight management program, supporting external validity. |
| Randomization | Participants randomly assigned | Because Intervention (INT) groups met earlier in the week than Control (CONT) groups, and the study had to begin by the end of June to permit the full 2 mo trial over summer, randomization was weighted toward INT at first then balanced with CONT in the final recruitment, to allow additional weekdays for baseline assessments prior to initiation of the program. Family units were enrolled in the following order: 6 INT, 1 CONT, 3 INT, 1 CONT, 1 INT, and 6 CONT. | All of the participants enrolled were included in the intention to treat analyses (with the exception of one parent who changed their mind following enrollment and consent/assent but prior to the first assessments). |
| Treatment allocation | 36 participants received treatment as allocated, by study group | Note that the fourth family unit (INT triad) had 2 Parent/Adult Caretakers (PAC), one of whom dropped out prior to baseline assessment, so did not receive treatment, but the remaining members of this unit continued. | Important counts for assessment of internal validity and interpretation of results; reasons for not receiving treatment as allocated should be given. |
| Follow-up | 36 participants completed treatment as allocated, by study group | Only 1 PAC participant did not complete treatment as allocated, as noted above under treatment allocation. | Bloodwork was incomplete (pre & post) on one CONT PAC who proved to be a difficult draw but overall excellent study adherence reassures the investigators of good internal validity and gives confidence in the interpretation of results. |
|  | 36 participants completed follow-up as planned, by study group | All participants completed follow-up as planned, by study group |  |
| Analysis | 36 participants included in main analysis, by study group | No participants were excluded from main analysis, by study group | The trial has been analyzed by intention to treat. |
