# Supplementary figures and images for: Randomized nutrient bar supplementation improves exercise-associated changes in plasma metabolome in adolescents and adult family members at cardiometabolic risk
Source: PLoS One. 2020 Oct 20;15(10):e0240437. doi: 10.1371/journal.pone.0240437 (PMC7575082; doi:10.1371/journal.pone.0240437)

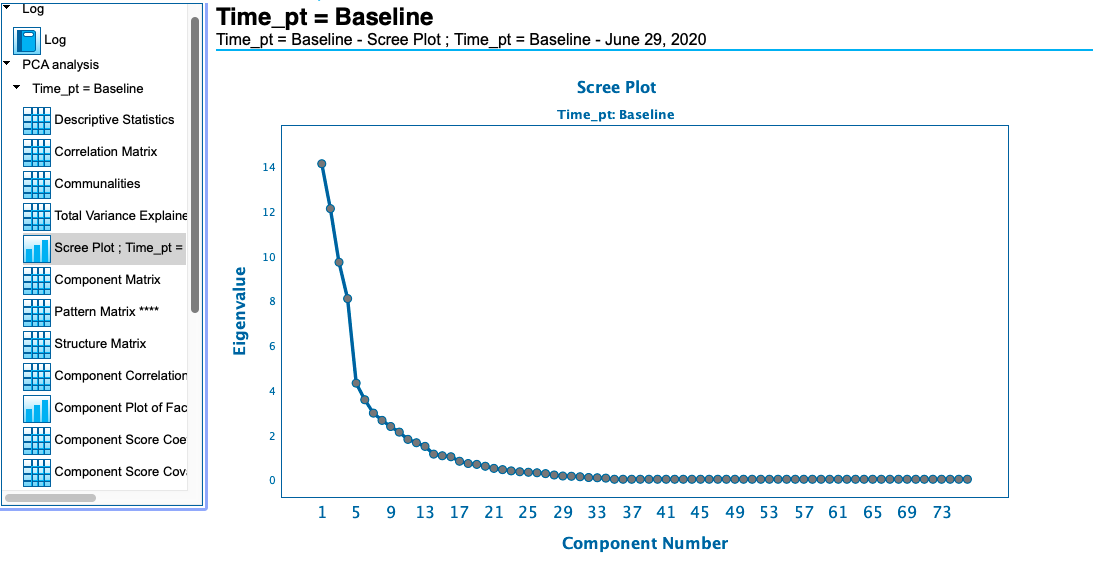

Supplement: S1 Fig — (TIFF) [file pone.0240437.s003.tiff]
