# Supplementary material for: Randomized nutrient bar supplementation improves exercise-associated changes in plasma metabolome in adolescents and adult family members at cardiometabolic risk
Source: PLoS One. 2020 Oct 20;15(10):e0240437. doi: 10.1371/journal.pone.0240437 (PMC7575082; doi:10.1371/journal.pone.0240437)
Supplement: Attachment — Submitted filename: PLOS One_Resubmission2ResponseToReviewers_24SEP2020.docx [file pone.0240437.s007.docx]

Sep 20, 2020

**Co-directors, Heart Institute**
*Charles I Berul, MD
Richard A Jonas, MD* **Chief, Division of Cardiology**
*Charles I Berul, MD* **Associate Chief, Division of Cardiology**
*Craig Sable, MD*

**Chief, Cardiovascular Surgery**
*Richard A Jonas, MD* **Director, Cardiac ICU**
*John T Berger, MD* **Director, Cardiac Anesthesia**
*Nina Deutsch, MD* **Administrative Director**
*Thomas S Wagner, MHA, FACHE*

**Cardiologists**
*Andrea Z Beaton, MD
Jeffrey A Becker, MD*
*Stanley D Beder, MD*
*John T Berger, MD*
*Charles I Berul, MD*
*Linda Bradley-Tiernan, MD*
*Kristin M Burns, MD
Sarah B Clauss, MD*
*Jessica H Colyer, MD
Russell R Cross, MD*
*Susan D Cummings, MD, MPH*
*Niti Dham, MD*
*Mary T Donofrio, MD*
*Robin W Doroshow, MD*
*Lowell H Frank, MD*
*Elena K Grant, MBChB*
*Ashraf S Harahsheh, MD*
*Deneen M Heath, MD
Thomas J Hougen, MD
Seiji Ito, MD
Anitha S John, MD, PhD
Jonathan R Kaltman, MD*
*Joshua P Kanter, MD
Sairah A Khan, MD
Darren Klugman, MD
Anita N Krishnan, MD
Linda Leatherbury, MD
Gerard R Martin, MD
Michele Mietus-Snyder, MD
Jeffrey P Moak, MD
Jai K Nahar, MD
Michael L O’Byrne, MD, MSCE
Laura J Olivieri, MD
Gail D Pearson, MD, ScD
David N Schidlow, MD, MMus
Elizabeth D Sherwin, MD
Christopher F Spurney, MD*

**Cardiac Surgeons**
*Richard A Jonas, MD
Dilip Nath, MD
Pranava Sinha, MD*

**Nursing Staff
Director, Inpatient Nursing**
*Lisa Williams, MHA, BSN, RNC, NE-BC*
**Clinical Manager, Ambulatory Services**
*Kristine Mehrtens, BSN, MS, RN, CPN*

Cover Letter for PLOS ONE revision2 PONE-D-34911

TITLE: Randomized Nutrient bar supplementation improves exercise-associated changes in plasma metabolome in adolescents and adult family members at cardiometabolic risk

Dear Dr. Ruscica,

Thank you for the encouraging response to our resubmission to PLOS One. We are grateful that reviewer 1 finds that all of comments have been addressed and that reviewer 3 appreciates the detailed respose to the initial critiques. Both still agree that this is a technically sound piece of research, presented intelligibly, and now both also agree that the statistical analyses have been performed appropriately and rigorously. Previously, all three reviewers agreed that we have been transparent with provision of the data on which our conclusions are based and we added more supplemental data (Block dietary FFQ) with revision 1. Therefore we are not sure why review 1 answered “No” this time to the same question as to whether all data underlying our findings have been made available. Please advise if specific additional data are requested and we will make every effort to oblige.

The single actionable comment that we respond to in this second revision came from Reviewer 3’s answer to Reviewer question 6 :

“I really appreciate the efforts to answer to my points. Detailed answered were provided, showing a deep knowledge on the statistical methods employed. Only one minor, but substantial point is still overlooked. The authors ackwonledge that the small sample size is a limitation of the study. However, all the employed tests are based on some assumptions (like the normality of the data, but not only) which must be checked to ensure a reliable inference. In the main text, I read "Continuous physical and metabolic variables were tested for normality by examining the skewness, kurtosis and the Shapiro Wilk tests and transformed as necessary before analysis". Please, provide evidence of the normality of the data and make clear which transformations are adopted and why.”

**RESPONSE:** In real-life datasets, variables do not always follow the normal distribution. All continuous physical and metabolic variables in our study were tested for normality. The results of the Shapiro-Wilk tests show that most of the variables in our data were normally distributed, but log transformations were conducted for the 11 of 31 continuous physical and metabolic variables that were skewed to make them as normal as possible. The results of the Shapiro-Wilk tests show (with *p*-value > 0.05) that all of the transformed variables except two (i.e. small and medium LDL particle concentrations) are approximately normal.

The measures of metabolite concentrations used for principal component analysis (PCA) were Z-transformed to render them normally distributed on the same scale with mean of zero and standard deviation of one. In addition, the generalized estimating equation (GEE) model used for our longitudinal data analysis is robust to data non-normality. The normality assumption of linear regression is about residuals [ε~ i.i.d. N(0, σ²)], and the GEE model does not necessarily assume normality of residuals.

We hope that this explanation will satisfy reviewer 3’s last remaining concern. We have added the following bolded wording (p10, lines 12-18) to clarify this important point:

"Continuous physical and metabolic variables were tested for normality by examining the skewness, kurtosis and the Shapiro Wilk tests and transformed as necessary before analysis." **Most of the variables in our data were normally distributed. Log transformations were conducted for the continuous physical and metabolic variables that were skewed to make them as normal as possible. The Shapiro-Wilk tests show (with *p*-value > 0.05) that all of the transformed variables except two (i.e. small and medium LDL particle concentrations) are approximately normal. The measures of metabolite concentrations used for principal component analysis (PCA) were Z-transformed to render them normally distributed on the same scale with mean of zero and standard deviation of one.**

We thank you for your prompt consideration. This manuscript will support a pending grant review if available for the study section to view when they meet mid-October.

Sincerely, on behalf of our authorship team,

Michele MIetus-Snyder MD
